# Chloroplast Stress Signals Orchestrate Epidermis‐Specific Remodeling of Mitochondria and ER Under High Light

**DOI:** 10.1002/advs.202514970

**Published:** 2026-01-27

**Authors:** Evan R. Angelos, Hee‐Seung Choi, Jingzhe Guo, Andrea A. Zanini, Tessa M. Burch‐Smith, Emily Snyder, Matthew Part, Wilhelmina van de Ven, Manhoi Hur, Gerd Ulrich Balcke, Alain Tissier, Quanqing Zhang, Katayoon Dehesh

**Affiliations:** ^1^ Institute For Integrative Genome Biology and Department of Botany and Plant Sciences University of California Riverside California USA; ^2^ Donald Danforth Plant Science Center St. Louis Missouri USA; ^3^ Department of Biology West Chester University of Pennsylvania West Chester Pennsylvania USA; ^4^ Department of Plant Biology University of California Davis California USA; ^5^ Department of Cell and Metabolic Biology Leibniz Institute of Plant Biochemistry Halle (Saale) Germany; ^6^ Proteomics Core Facility Institute For Integrative Genome Biology and Department of Botany and Plant Sciences University of California Riverside California USA

**Keywords:** chloroplast signaling, epidermal organelle dynamics, high light stress, mitochondrial‐ER remodeling, Miro1, organelle plasticity

## Abstract

Environmental stress demands precise coordination among organelles to maintain cellular homeostasis. In *Arabidopsis*, high light (HL) exposure triggers chloroplast‐dependent remodeling of mitochondrial and endoplasmic reticulum (ER) morphology specifically in adaxial and abaxial epidermal cells, but not in mesophyll cells. Live‐cell imaging reveals that HL rapidly suppresses mitochondrial motility, followed by fusion‐driven elongation and ER cisternal expansion. Inhibition of photosynthetic, but not mitochondrial, electron transport abolishes these changes, confirming chloroplast activity as the upstream trigger. Pharmacological analyses show that exogenous H_2_O_2_ induces mitochondrial elongation, whereas calcium chelation blocks both H_2_O_2_‐ and HL‐induced responses, demonstrating that chloroplast‐derived H_2_O_2_ activates a Ca^2^^+^ flux essential for remodeling. Proteomic and functional studies identify the Ca^2^^+^‐binding GTPase MIRO1 as a central integrator of this pathway. MIRO1 overexpression mimics HL‐induced morphodynamics, while mutations disrupting its Ca^2^^+^‐binding or acetylation motifs abolish the response, establishing Ca^2^^+^‐dependent MIRO1 activity as a prerequisite for remodeling. Together, these findings reveal an epidermis‐specific, light‐responsive network in which chloroplast‐derived H_2_O_2_ initiates Ca^2^^+^ signaling through MIRO1 to coordinate mitochondrial and ER remodeling—a spatially restricted mechanism of organellar communication and stress adaptation at the plant–environment interface.

## Graphical abstract

High light exposure triggers an epidermis‐specific remodeling of mitochondria and ER in *Arabidopsis*, driven by chloroplast‐derived signals. Live‐cell imaging shows that HL rapidly suppresses mitochondrial motility, followed by fusion‐driven elongation and ER cisternal expansion. MIRO1 emerges as a key mediator, linking chloroplast activity to dynamic interorganellar restructuring at the plant–environment interface.

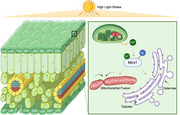

## 1 Introduction

Plants constantly encounter environmental fluctuations that impose metabolic and structural perturbations, necessitating dynamic adaptations in organelle morphodynamics to sustain cellular homeostasis. A fundamental consequence of these stressors is the disruption of plastidial metabolism, which serves as a metabolic hub. In response to stress, plastids communicate with other cellular compartments through metabolites, signaling molecules, physical contact sites, and retrograde signaling pathways that coordinate organelle‐wide stress responses [1, 2, 3, 4, 5], and retrograde signaling pathways that coordinate organelle‐wide responses and influence the functional integrity of both mitochondria and the endoplasmic reticulum (ER) [6, 7, 8, 9]. Indeed, disruptions in plastidial function have been shown to significantly reconfigure mitochondrial metabolism, emphasizing a strong functional interdependence between these organelles [10].

High light (HL) is a prominent environmental stressor that perturbs photosynthetic homeostasis by generating excess excitation energy, affecting both chloroplast and mitochondrial function. Mitochondria play a supporting role under HL by facilitating photorespiration and dissipating excess reducing equivalents via the import of chloroplast‐derived malate through the malate valve [11, 12]. These functions reflect a tight metabolic interdependence between plastids and mitochondria, further emphasized by observations that plastidial dysfunction can dramatically reconfigure mitochondrial metabolism

Mitochondria are inherently dynamic organelles that transition between fission and fusion states in response to fluctuations in metabolic demand and oxidative stress [13, 14, 15, 16, 17]. In mammalian systems, stress‐induced fission, mediated by Dynamin Related Protein 1 (DRP1) and Fission 1 (FIS1), segregates damaged mitochondrial components into smaller units targeted for mitophagy, thereby preventing the release of pro‐apoptotic factors [17, 18, 19, 20]. In contrast, fusion, facilitated by Mitofusin protein 1/2 (MFN1/2) and Optic Atrophy Protein 1 (OPA1), enables content mixing, diluting damaged components, enhancing oxidative phosphorylation, and promoting stress resilience [19, 21, 22, 23]. These processes are tightly regulated by post‐translational modifications (e.g., acetylation, ubiquitination, phosphorylation) and by physical interactions with the ER and actin cytoskeleton [17, 24, 25, 26, 27, 28]. ER and actin are essential at most mitochondria fusion sites [29, 30]. ER tubules also initiate fission through the formation mitochondria‐ER contact sites (MERCs) where they recruit actin motor proteins to promote mitochondrial constriction [31, 32, 33, 34, 35]. Disruption of these regulatory mechanisms is implicated in the pathogenesis of disorders such as Parkinson's, Alzheimer's, and non‐alcoholic fatty liver disease [36].

While the principle of dynamic fission and fusion in response to cellular stress is likely conserved in plants [15, 16, 37, 38, 39], the underlying molecular components and regulatory mechanisms remain less well characterized. Reverse genetics has uncovered conserved components of the mitochondrial fission machinery in *Arabidopsis*, including DRP3A/B (orthologs of DRP1) and FIS1A/B (FIS1 orthologs). However, despite multiple forward genetic screens, the molecular machinery responsible for mitochondrial fusion in plants remains unidentified [14, 39, 40, 41, 42, 43, 44]. This raises the possibility that plant‐specific proteins, such as Elongated Mitochondria 1(ELM1) [39], or conserved proteins with newly adapted functions may regulate mitochondrial fusion dynamics. Furthering this idea, both ER and mitochondrial remodeling events have been frequently observed in plant cells [44, 45, 46]. Yet, how these two organelles coordinate their structural adaptations, particularly in response to environmental stress, remains poorly understood.

This study identifies a chloroplast‐driven mechanism of organelle communication in plants, where HL triggers epidermis‐specific remodeling of mitochondria and the ER. Chloroplast activity, rather than mitochondrial electron transport, initiates fusion‐driven mitochondrial elongation and ER reorganization, coordinated by the GTPase MIRO1. Proteomic and functional analyses reveal that MIRO1's acetylation and calcium‐binding domains are essential for this process. These findings uncover a tissue‐specific pathway of plastid control over cellular architecture, reflecting conserved principles of interorganellar communication.

## 2 Results

### 2.1 High Light Induces Epidermis‐Specific Mitochondrial Elongation

The Methylerythritol Phosphate (2‐C‐methyl‐D‐erythritol 4‐phosphate; MEP) pathway, a plastid‐localized isoprenoid biosynthesis pathway comprising seven enzymes, plays a pivotal role in isoprenoid biosynthesis, supplying critical precursors for plasma membrane integrity, signaling molecules, pigments, and protein prenylation (Figure S1A) [47, 48, 49, 50]. This pathway is also integral to plastidial redox homeostasis and interorganellar communication [3]. Among its intermediates, 2‐C‐methyl‐D‐erythritol‐2,4‐cyclodiphosphate (MEcPP) functions as a known retrograde signaling metabolite, linking plastidial metabolic status to nuclear gene expression and broader cellular responses including significant alterations to mitochondrial and ER dynamics [6, 7, 8, 9, 51, 52]. To investigate the effects of MEP pathway disruption, we generated DEX‐inducible RNAi lines targeting DXS, HDS, and HDR, key enzymes in this pathway. Chlorotic leaf emergence was evident 72 h post‐DEX induction in RNAi lines but not in controls, indicating a functional perturbation of plastidial metabolism (Figure S1B).

To assess how MEP pathway perturbation affects mitochondrial metabolism, we analyzed tricarboxylic acid (TCA) cycle metabolites in wild‐type (WT) and RNAi plants. This revealed differential shifts in metabolite levels, indicating a rewiring of mitochondrial metabolic flux in response to MEP disruption (Figure S1C). Given that mitochondrial morphology is closely linked to metabolic state [17, 53], we next examined whether these metabolic shifts influence mitochondrial morphology.

Initial mitochondrial imaging using MitoTracker Red (MTR) was hampered by MTR‐induced artifacts, which exaggerated mitochondrial size, possibly due to potential non‐specific binding or altered membrane potential [54, 55] (Figure S1D). To eliminate these artifacts, we introgressed the 3x‐HA‐sGFP‐TOM5 [56] (hereafter GFP‐TOM5) reporter, into our RNAi lines, allowing for direct visualization of mitochondrial membranes (Figure S1E). Using this approach, we confirmed a significant increase in mitochondrial cross‐sectional area across all RNAi lines 72 h post‐DEX induction (Figure S1F, Table S1), reinforcing the link between MEP pathway activity and mitochondrial morphology, albeit independent of the accumulation of specific higher‐order intermediates.

To determine how perturbation of the MEP pathway influences mitochondrial dynamics under a prevalent environmental stress, light stress (HL), mitochondrial morphology was examined in HDSi plants treated with DEX or mock solution and maintained under standard light conditions for 66 h, followed by 6 h of HL or continued standard light exposure (Figure S1G,H). Under standard light, DEX treatment alone showed no impact on mitochondria size in the third leaf but a modest mitochondrial elongation in the fifth leaf coinciding with visible chlorosis caused by transgene induction. When DEX‐treated plants were exposed to HL, mitochondria exhibited elongation in both the third and fifth leaves; however, the extent of elongation was considerably lower in the fifth leaf, likely due to its compromised physiological state. The reduced elongation in chlorotic tissue likely reflects diminished mitochondrial fusion activity and impaired energy metabolism, resulting from the combined effects of MEP pathway suppression and HL‐induced photooxidative stress. These findings indicate that MEP pathway activity modulates mitochondrial morphology in a light, where DEX‐induced chlorosis attenuates HL‐driven mitochondrial elongation by limiting the metabolic and redox capacity necessary to sustain mitochondrial dynamics.

Building on these findings, and recognizing that HL stress modulates MEP pathway activity [51, 57], we next investigated the broader effects of HL exposure on mitochondrial morphology, using two independent mitochondrial marker lines: GFP‐TOM5 [56], an outer membrane‐associated marker, and *SHMT‐roGFP2‐Grx1* [58] (hereafter SHMT‐roGFP2), a soluble mitochondrial matrix marker (Figure S2A). Both marker lines exhibited significant mitochondrial elongation under HL conditions (Figure S2B), demonstrating that mitochondrial elongation is independent of GFP localization, however SHMT‐roGFP2 was selected for use in further morphology experiments due to brightness of the fluorescence signal. Additionally, while a direct comparison between MTR‐stained mitochondria and SHMT‐roGFP2 confirmed that MTR significantly overestimated mitochondrial elongation, HL treatment led to further elongation of MTR‐stained samples (Figure S2C,D). These findings demonstrate that HL‐induced mitochondria elongation is not an artifact of GFP marker overexpression, while underscoring the importance of using genetically encoded markers for accurate mitochondrial morphology analyses.

To determine whether specific light wavelengths influence mitochondrial elongation, we examined standard light (SL), and three different HL conditions enriched in different wavelengths (HL #1, HL #2, HL #3) and found no significant differences between high lights (Figure S2E,F).

**FIGURE 1:**
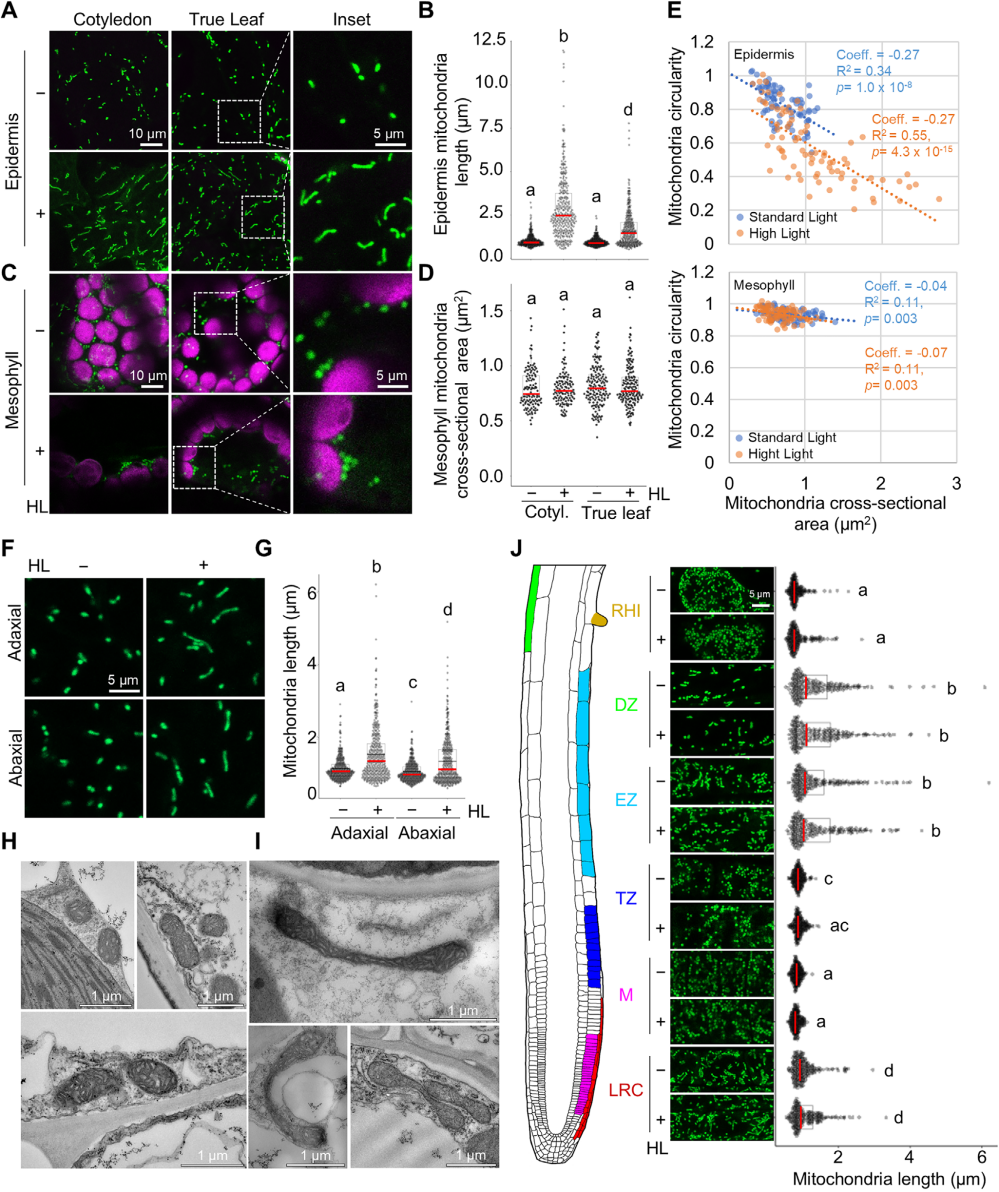
Tissue‐Specific Alterations in Mitochondrial Morphology in Response to High Light (HL) Exposure. (A–D) Representative images and quantification of mitochondrial length in epidermal (A,B) and mitochondria cross sectional area in mesophyll (C,D) tissues of cotyledons and true leaves in transgenic plants expressing SHMT‐roGFP2 under high light (HL+) and standard light (HL−) conditions. Magnified insets of regions outlined by dotted white lines are shown. (D). Letters in (B and D) indicate statistically distinct groups determined by two‐way weighted least squares ANOVA (hereafter wANOVA) and Tukey's post‐hoc test (see Table S1 full results of statistical analyses). Red lines on box and whisker plots denote median value, for (B) *n* = 320 mitochondria; for (D) *n* = 120–160 mitochondria (see Table S1 Data Summary for extended data description of all imaging datasets); α = 0.05. (E) Comparison of mitochondrial circularity and cross‐sectional area in epidermal and mesophyll cells under standard and high light conditions; *n* = 80. Color‐coded dashed lines and corresponding linear regression statistics for each treatment condition are shown. (F–G) Representative confocal images and quantification of mitochondrial length in adaxial and abaxial epidermal cells. Scale bar = 10 µm. Letters denote statistically distinct groups: two‐way wANOVA; Tukey's post‐hoc test; *n* = 400 mitochondria; α = 0.05. (H,I) Transmission electron micrographs of representative mitochondria under standard light (H) and high light (I) conditions. The upper panel in (I) shows a micrograph from a 150 nm section; all other images in (H,I) are from 70 nm sections. (J) Representative images and quantification of mitochondrial length across root zones, as illustrated in the schematic: root cap (RC), meristematic zone (M), transition zone (TZ), elongation zone (EZ), differentiation zone (DZ), and root hair initiation sites (RHI) under high light (HL+) and standard light (HL−) conditions. Letters denote statistically distinct groups: two‐way wANOVA; Tukey's post‐hoc test; *n* = 200 mitochondria; α = 0.05.

We next examined tissue‐specific responses to HL‐induced mitochondrial elongation (Figure 1A). In leaf epidermal cells, HL exposure led to a significant increase in mitochondrial length (Figure 1B). In contrast, mitochondria in mesophyll cells remained predominantly rounded in shape, with no significant change in size (Figure 1C,D). Quantitative analysis of mitochondrial circularity and cross‐sectional area revealed distinct tissue‐specific morphological patterns. In epidermal cells, a strong inverse correlation between these parameters indicates elongation‐driven extension. In contrast, mesophyll mitochondria exhibited a weak correlation, suggesting that differences in size were primarily due to radial expansion (Figure 1E).

To assess whether the limited light penetration through tissue layers might constrain mitochondrial elongation in mesophyll cells, or whether these cells exhibit an inherently distinct response to HL, we illuminated leaves from above and examined mitochondrial morphology across adaxial, abaxial, and mesophyll cell layers. Within the epidermis, both adaxial and abaxial cells displayed HL‐induced mitochondrial elongation, though the effect was more pronounced in the adaxial layer (Figure 1F,G). Because illumination was applied from above, light intensity was highest at the upper epidermis but remained sufficient to reach the lower surface, enabling detectable elongation of mitochondria in abaxial cells, albeit to a lesser extent. Despite this light penetration, HL exposure did not elicit measurable changes in mitochondrial morphology within the mesophyll, indicating that the response is specific to epidermal cells rather than a general feature of all illuminated tissues.

These epidermal morphologies, initially observed by confocal microscopy, were further validated by TEM analysis, which confirmed the prevalence of elongated, dumbbell‐shaped, or tubular mitochondria following HL exposure (Figure 1H,I).

To determine whether HL influences mitochondrial morphology in non‐photosynthetic tissues, we examined mitochondria in epidermal cells across different root developmental zones under SL and HL conditions (Figure 1J). Mitochondria length was independent of light exposure across developmental zones; however, mitochondria were significantly longer in the elongation zone (EZ) and differentiation zone (DZ), except for root hair initiation (RHI) sites within the DZ, suggesting that mitochondrial morphology correlates with physiological demands of specific developmental stages in root tissues, rather than light exposure.

In summary our findings establish that HL stress selectively induces mitochondrial elongation in leaf epidermal cells, independent of light wavelength, highlighting a fundamental tissue‐specific difference in mitochondrial morphodynamics, with epidermal mitochondria and not mesophyll mitochondria, undergoing elongation.

### 2.2 High Light‐Induced Reduction in Mitochondrial Motility Precedes Increased Mitochondrial Fusion

**FIGURE 2:**
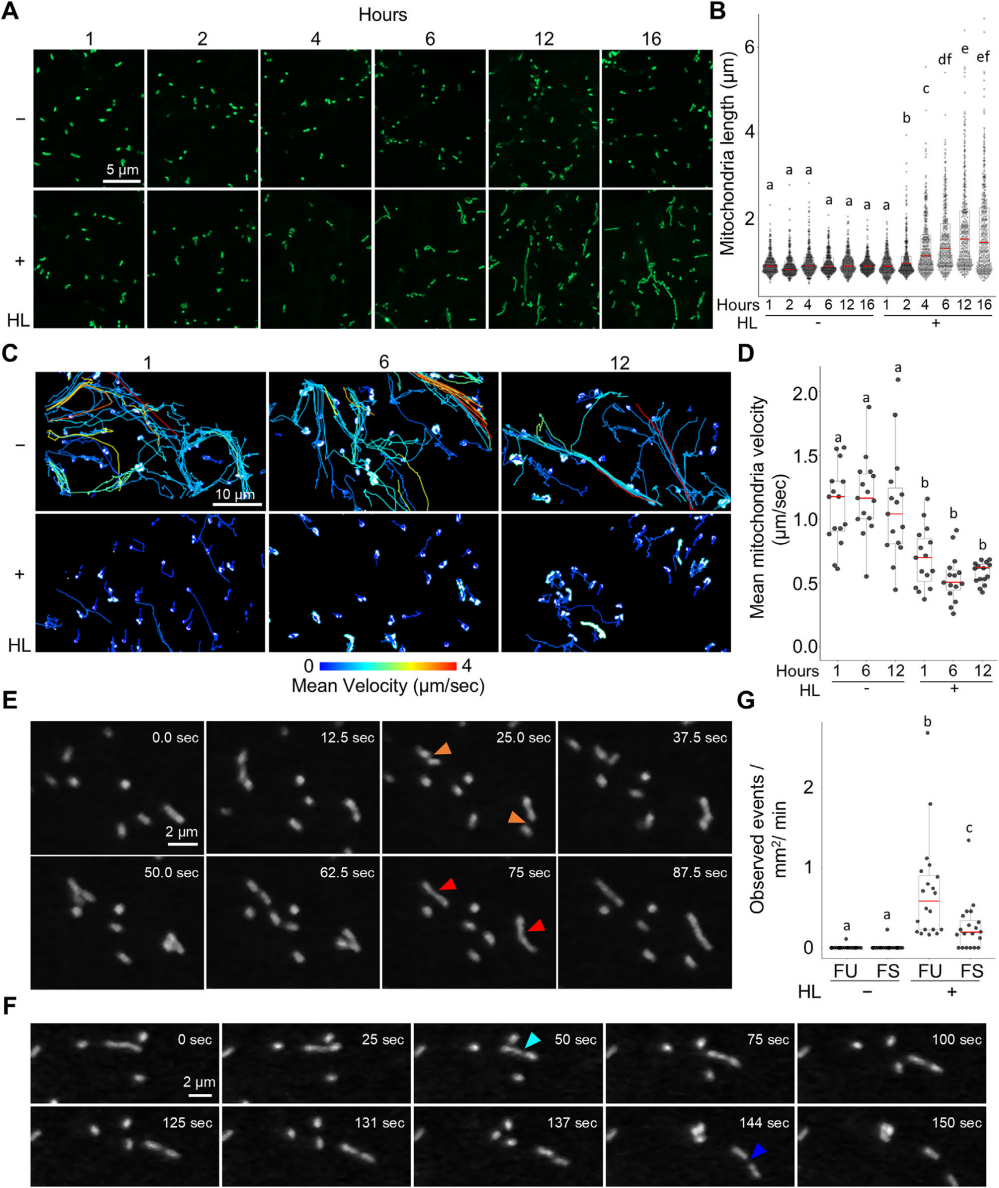
High Light‐Induced Reduction in Mitochondrial Motility Precedes Increased Mitochondrial Fusion. (A,B) Time‐course analysis of mitochondrial elongation in plants exposed to high light (HL+) for 1, 2, 4, 6, 12, and 16 h, compared to standard light (HL−). Letters denote statistically distinct groups: two‐way wANOVA; Tukey's post‐hoc test; *n* = 320 mitochondria; α = 0.05. (C,D) Representative images and quantification of mitochondrial movement over time, with each track color‐coded by mean velocity. Mean mitochondrial velocity (µm/s) of all mitochondria in an individual recording was quantified at 1‐, 6‐, and 12 h post‐HL treatment. Letters denote statistically distinct groups: two‐way wANOVA; Tukey's post‐hoc test; *n* = 15 recordings; α = 0.05. (E–G) Representative images and quantification of mitochondrial fusion (FU) (E) and fission (FS) (F).Orange and red arrows indicate the initiation and conclusion of fusion events, respectively, while cyan and blue arrows mark the initiation and conclusion of fission events. Quantification in (G) shows fission and fusion events, normalized to observed area and recording length. Letters denote statistically distinct groups: two‐way wANOVA; Tukey's post‐hoc test; *n* = 20 recordings; α = 0.05.

To better understand HL‐induced changes in mitochondrial dynamics, we conducted a time‐course analysis of mitochondrial elongation at 1, 2, 4, 6, 12, and 16 h post‐HL exposure. Mitochondrial elongation was first evident between 2–4 h and peaked at 12 h (Figure 2A,B). To further characterize mitochondrial behavior under HL conditions, we performed kymograph‐based tracking of individual mitochondria at 1, 6, and 12 h post‐treatment to assess changes in motility. Representative kymographs and trajectory maps, color‐coded by mean velocity, illustrate a marked reduction in mitochondrial movement following HL exposure. (Figure 2C,D; Movie S1). Quantitative and statistical analysis revealed a significant decrease in mitochondrial motility within the first hour, reaching its lowest point at 6 h (Figure 2D). Notably, this reduction in motility precedes mitochondrial elongation, suggesting that decreased organelle movement is a tightly regulated aspect of the stress response and may be a prerequisite for elongation. To assess whether changes in mitochondrial morphology reflect altered dynamics, we quantified fusion (FU) and fission (FS) events using spinning disc confocal microscopy, which provides high temporal resolution for capturing these processes. Fusion and fission frequencies were normalized to the observed area and recording duration. After 12 h of HL exposure, we observed a significant increase in the FU/FS ratio (Figure 2E–G; Movie S2), indicating a shift toward mitochondrial network integration.

**FIGURE 3:**
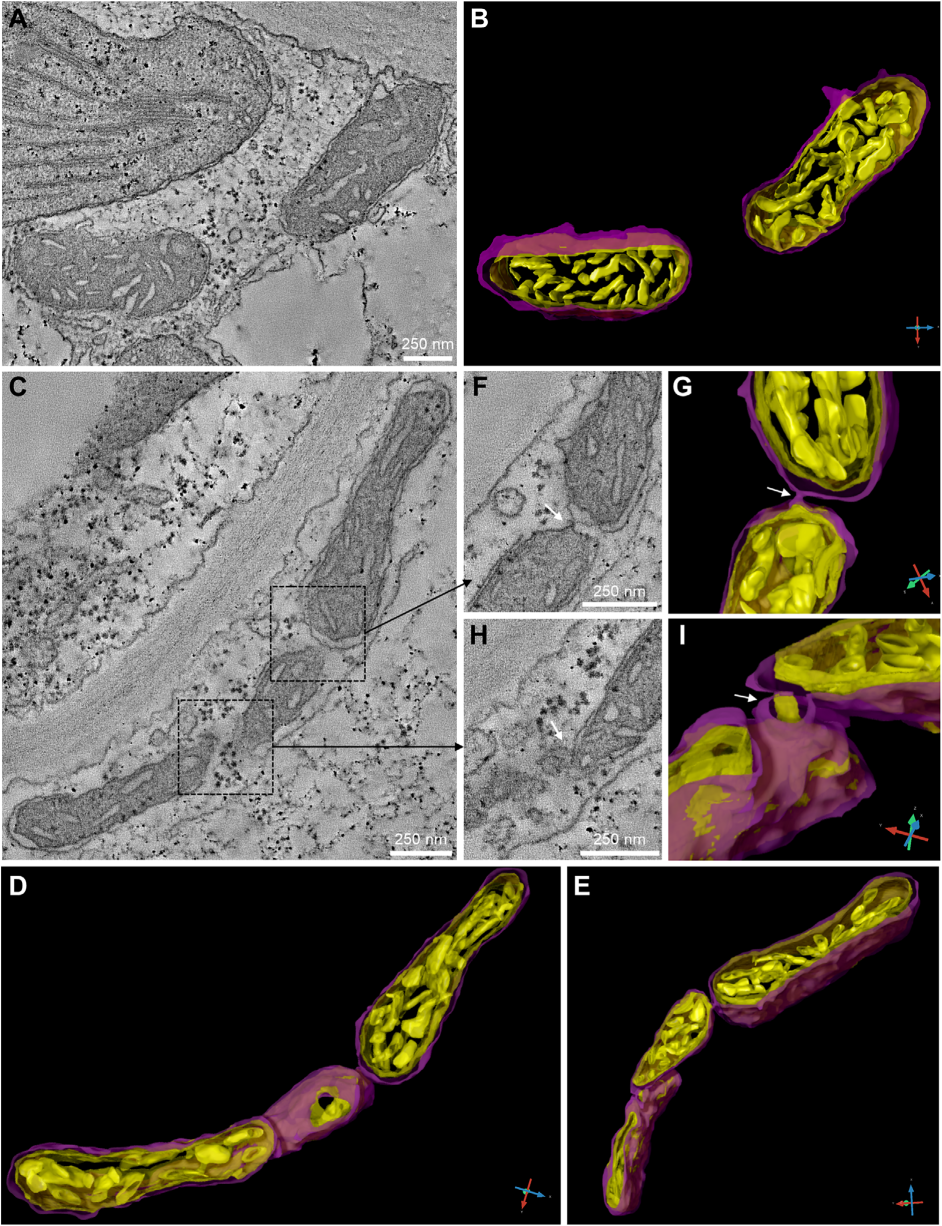
Transmission electron microscopy (TEM) and tomographic analyses of mitochondrial ultrastructure under standard and high light conditions. (A,B) TEM image (frame 53 from tomogram in Movie S3) and corresponding 3D reconstruction of mitochondria in epidermal cells exposed to standard light, showing typical ultrastructure. (C–E) TEM image (frame 104 from tomogram in Movie S5) and corresponding 3D reconstructions of mitochondria in epidermal cells exposed to high light, showing three mitochondria approaching at their ends. (F–I) TEM images captured at different tilt angles (frame 168 for F and frame 158 for H, of the tomogram in Movie S5) and their respective 3D reconstructions of mitochondria of epidermal cells exposed to high light conditions. White arrows indicate points of contact between adjacent mitochondria. Scale bars in TEM images represent 250 nm. Color code for 3D reconstructions B, D, E, G, I: magenta indicates outer envelope membrane; yellow, is the inner membrane and cristae. The arrows at the corners show the orientation of the *x* (blue), *y* (red), and *z* (green) axes.

Complementary ultrastructural analyses using transmission electron microscopy (TEM) and electron tomography confirmed HL‐induced mitochondrial remodeling. Under standard light, mitochondria appeared as discrete organelles with minimal proximity or interaction (Figure 3A,B; Movies S3 and S4). In contrast, HL‐treated cells showed elongated mitochondria with frequent close appositions. Tomograms revealed stable membrane contact sites between adjacent mitochondria, including parallel outer membrane alignment and occasional tether‐like structures (Figure 3C–I; Movies S5 and S6), suggesting that HL promotes physical interactions that facilitate mitochondrial fusion and network formation.

Together, these results suggest that HL stress triggers a sequential mitochondrial response: a rapid decline in motility, the establishment of physical contact sites, and a subsequent increase in fusion and elongation, potentially optimizing energy production and buffering metabolic stress under prolonged HL conditions.

### 2.3 High Light‐Induced Mitochondrial Elongation Requires Photosynthetic Electron Transport

**FIGURE 4:**
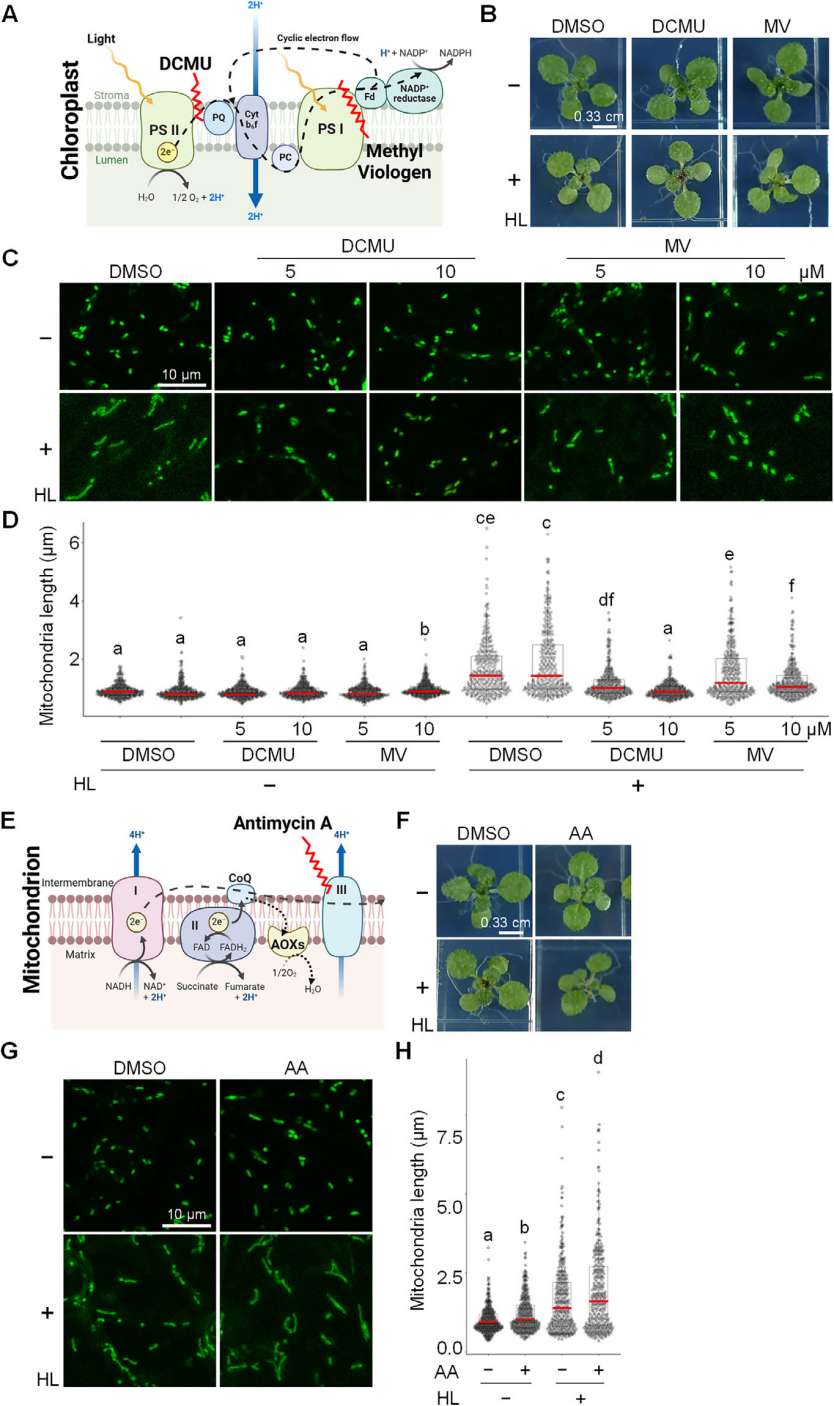
High Light‐Induced Mitochondrial Elongation Requires Functional Photosystems but Not Mitochondrial Electron Transport Chain Activity. (A) Schematic representation of the action sites of 3‐(3,4‐Dichlorophenyl)‐1,1‐dimethylurea (DCMU) and Methyl viologen (MV), which inhibit PSII and PSI, respectively. (B) Representative images of plant phenotypes treated with dimethyl sulfoxide (DMSO) as a vehicle or the photosystem inhibitors DCMU and MV, followed by 12 h of high light (HL+) or standard light (HL−) exposure. Scale bar: 0.33 cm. (C,D) Representative confocal images and quantification of mitochondria length in leaf epidermal cells exposed to standard light (HL‐) or high light (HL+) for 12 h, with or without treatment with DMSO, DCMU, or MV. Scale bar: 10 µm. Letters denote statistically distinct groups: two‐way wANOVA; Tukey's post‐hoc test; *n* = 320 mitochondria; α = 0.05. (E) Schematic representation of the action sites of antimycin A (AA), an inhibitor of mitochondrial complex III. (F) Representative images of plant phenotypes treated with DMSO (control) or the mitochondrial electron transport inhibitor AA, followed by exposure to high light (HL+) or standard light (HL−) for 12 h. Scale bar: 0.33 cm. (G,H) Representative confocal images and quantification of mitochondria length in leaf epidermal cells exposed to standard light (HL‐) or high light (HL+) for 12 h, with or without treatment with DMSO or AA. Scale bar: 10 µm. Letters denote statistically distinct groups: two‐way wANOVA; Tukey's post‐hoc test; *n* = 320 mitochondria; α = 0.05.

To investigate the regulatory factors controlling HL‐induced mitochondrial elongation and given the central role of photosynthetic electron flow in plant energy metabolism, we tested whether disrupting Photosystem II (PSII) with DCMU (3,4‐Dichlorophenyl)‐1,1‐dimethylurea) and Photosystem I (PSI) with methyl viologen (MV) would affect this process (Figure 4A). Although plants treated with either inhibitor and exposed to HL for 12 h showed no visible phenotypic differences compared to the DMSO control (Figure 4B), confocal imaging of leaf epidermal cells revealed striking differences in mitochondrial morphology. Mitochondrial elongation observed in DMSO‐treated plants under HL was completely abolished by DCMU and strongly suppressed by MV (Figure 4C,D). These findings indicate that photosynthetic electron transport is a key upstream regulator of HL‐induced mitochondrial dynamics.

To evaluate whether mitochondrial electron transport chain (ETC) activity contributes to HL‐induced mitochondrial elongation, we first confirmed the effectiveness of antimycin A (AA), a Complex III inhibitor (Figure 4E), using a cytosolic Peredox‐mCherry NADH/NAD^+^ sensor line [59]. AA treatment under both standard light and HL increased the NADH/NAD^+^ ratio, however, the combination of HL and AA did not produce an additive effect (Figure S3A,B). This is consistent with previous findings indicating an upper limit to the NADH/NAD^+^ ratio in plants exposed to light while treated with AA [59]. To further validate ETC inhibition under HL, plants were transferred to darkness for 6 h following 12 h of HL exposure. Darkness significantly reduced the NADH/NAD^+^ ratio in HL+DMSO plants but had no effect on HL+AA plants, confirming that AA effectively blocks ETC activity under both light conditions (Figure S3B).

Despite confirmed ETC inhibition, AA‐treated plants showed no visible phenotypic differences (Figure 4F), and mitochondrial elongation under HL remained unaffected, as mitochondrial lengths in HL+AA plants were comparable to those in HL+DMSO controls (Figure 4G,H; Figure S3C,D).

Together, these results demonstrate that HL‐induced mitochondrial elongation is independent of mitochondrial ETC activity and is instead primarily driven by photosynthetic energy metabolism.

Given our metabolite measurements showing increased levels of TCA cycle intermediates, specifically fumarate (FUM) and malate (MAL), following HL treatment (Figure S4A), we next tested whether their accumulation could independently induce mitochondrial elongation through exogenous application. However, confocal imaging and subsequent quantification revealed no morphological differences between FUM/MAL‐treated plants and mock controls (Figure S4B,C). These results indicate that the accumulation of TCA cycle intermediates is insufficient to trigger mitochondrial elongation, supporting the conclusion that this response is primarily regulated by photosynthetic electron transport, rather than mitochondrial respiration or metabolic flux.

Collectively, our findings show that HL‐induced mitochondrial elongation depends on functional photosystems but occurs independently of mitochondrial ETC activity, cytosolic reducing equivalent accumulation, or TCA intermediate levels. This suggests that mitochondrial morphological remodeling under HL may be mediated by alternative signals, such as reactive oxygen species (ROS), calcium fluxes, or redox changes associated with photosynthetic activity.

### 2.4 H_2_O_2_‐Induced Mitochondrial Elongation Requires Intracellular Ca^2^^+^

**FIGURE 5:**
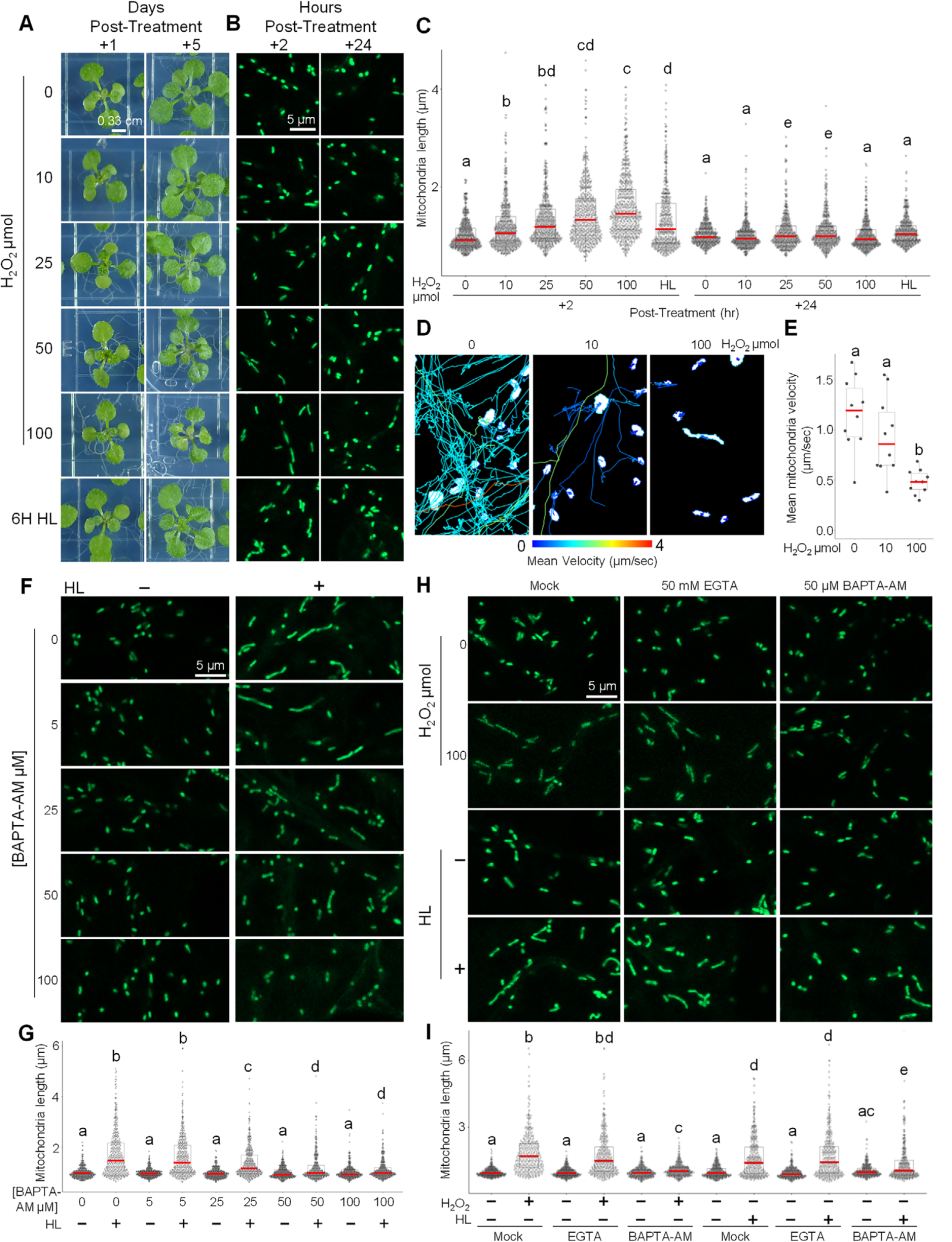
Reversible mitochondrial elongation induced by H_2_O_2_ or high light requires intracellular calcium. (A) Representative images of plants sprayed with the indicated amounts of H_2_O_2_ (µmol per 100 mm × 100 mm Petri dish; see Materials and Methods) or exposed to 6 h of high light (HL). Plants were then returned to standard light conditions for 1 or 5 days to assess post‐stress viability. Scale bar = 0.33 mm. (B–C) Representative confocal images and quantification of mitochondrial length measured at 2 and 24 h post‐treatment with the indicated amounts of H_2_O_2_ or following 6 h of high light (HL) exposure. Scale bar = 5 µm. Letters denote statistically distinct groups: two‐way wANOVA; Tukey's post‐hoc test; *n* = 400 mitochondria; α = 0.05. (D,E) Representative confocal images and quantitative analyses of mitochondrial movement showing a progressive reduction in motility over time after treatment with increasing quantities of H_2_O_2_. Each trajectory is color‐coded by mean velocity. Mean mitochondrial velocity (µm s^−^^1^) of all mitochondria in an individual recording was quantified 2 h after treatment with 0, 10, or 100 µmol H_2_O_2_ per plate. Letters denote statistically distinct groups: one‐way ANOVA; Tukey's post‐hoc test; n = 10 recordings; α = 0.05. (F,G) Representative confocal images and quantification of mitochondrial length in plants pretreated with the cell‐permeable calcium chelator BAPTA‐AM for 2 h, then subjected to HL or maintained under standard light for 6 h. Scale bar = 5 µm. Letters denote statistically distinct groups: two‐way wANOVA; Tukey's post‐hoc test; *n* = 400 mitochondria; α = 0.05. (H,I) Representative confocal images and quantification of mitochondrial length in plants pretreated with either BAPTA‐AM or the cell‐impermeable calcium chelator EGTA for 2 h, then treated with 100 µmol H_2_O_2_ or exposed to 6 h of HL. Scale bar = 5 µm. Letters denote statistically distinct groups: two‐way wANOVA; Tukey's post‐hoc test; *n* = 320 mitochondria; α = 0.05.

To test whether chloroplast‐derived H_2_O_2_ mediates mitochondrial elongation under HL, we examined the effects of exogenous H_2_O_2_ on mitochondrial morphology. Spraying plants with increasing concentrations of H_2_O_2_ (10–100 µmol per plate) induced visible chlorotic patches at the highest concentration but did not compromise overall plant viability over five days (Figure 5A). Confocal imaging revealed a clear, dose‐dependent mitochondrial elongation 2 h after treatment, resembling the response observed following HL exposure (Figure 5B,C). Mitochondrial morphology returned to baseline by 24 h after treatment (Figure 5B,C), indicating that the response is reversible.

To determine whether this response is specifically driven by H_2_O_2_ rather than generalized oxidative stress, plants were pretreated with various antioxidants, including ascorbic acid (AsA), catalase (CAT), reduced glutathione (GSH), and N‐acetylcysteine (NAC), and sorbitol (negative control). None of these treatments altered HL‐induced mitochondrial elongation (Figure S5). The lack of antioxidant effect may reflect several factors: exogenous antioxidants may not efficiently reach or neutralize localized, transient H_2_O_2_ bursts at chloroplast–mitochondria contact sites, or the critical signaling window may occur before they can act. Additionally, limited cell permeability and compartmentalization may restrict antioxidant access to relevant subcellular sites, while endogenous buffering or compensatory ROS generation could maintain sufficient signaling despite treatment.

Kymograph‐based tracking further revealed that increasing H_2_O_2_ concentrations progressively suppressed mitochondrial motility (Figure 5D,E), consistent with a shift toward fusion‐driven elongation. To assess whether Ca^2^^+^ signaling mediates this process, seedlings were pretreated with the Ca^2^^+^ chelator BAPTA‐AM. Chelation with BAPTA‐AM abolished and HL‐induced mitochondrial elongation in a concentration‐dependent manner (Figure 5F,G). H_2_O_2_ or HL applied to mock treated plants consistently induced mitochondrial elongation (Figure 5H,I). Likewise, co‐application of HL with EGTA or H_2_O_2_ with EGTA, a cell‐impermeable Ca^2^^+^ chelator, did not alter this elongation response. In contrast, co‐treatment with BAPTA‐AM, the cell‐permeable Ca^2^^+^ chelator, completely suppressed mitochondrial elongation induced by either HL or exogenous H_2_O_2_ (Figure 5H,I).

Together, these findings demonstrate that H_2_O_2_ is sufficient to induce transient mitochondrial elongation and that this process requires intracellular Ca^2^^+^, establishing a functional link between H_2_O_2_ signaling and Ca^2^^+^‐dependent mitochondrial morphodynamics.

### 2.5 Epidermal Mitochondrial Proteome of High Light‐Treated Plants Identifies Mitochondrial‐ER Interface Proteins

**FIGURE 6:**
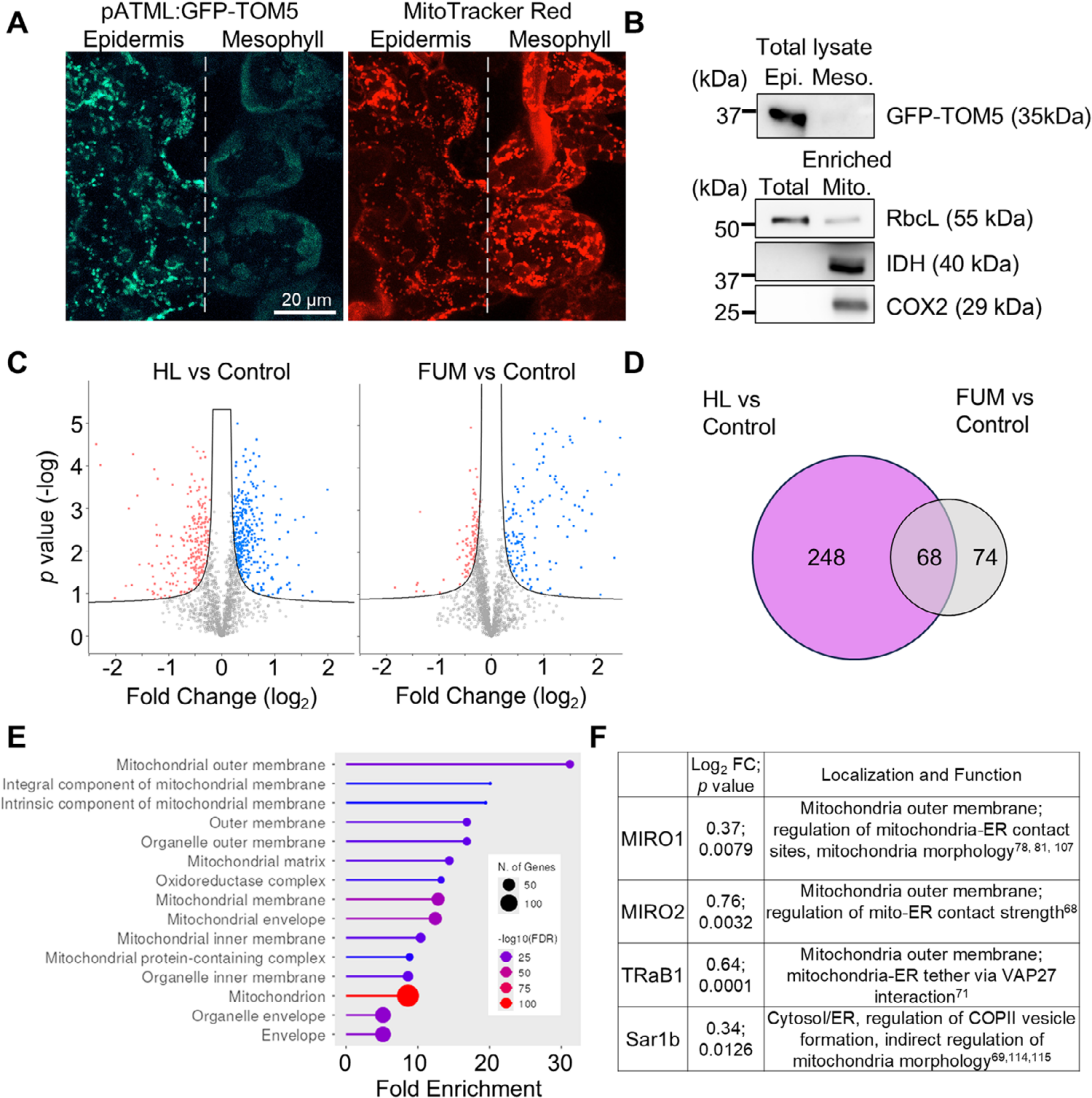
Epidermal Mitochondrial Proteome of High Light‐Treated Plants. (A) Representative confocal images of mitochondria in a transgenic line expressing GFP‐TOM5 under the epidermis‐specific promoter pATML1 show GFP fluorescence exclusively in epidermal tissues. In contrast, MitoTracker Red staining reveals a similar mitochondrial density in both epidermal and mesophyll cells, further confirming the tissue specificity of pATML1:GFP‐TOM5 expression. (B) Western blot analysis of mitochondrial proteins isolated from epidermal peels and mesophyll tissues of *pATML1:GFP‐TOM5* plants using GFP antibodies. Total lysate and enriched mitochondrial fractions were probed with antibodies against RbcL, IDH, and COX2 to validate mitochondrial enrichment. (C) Volcano plots depicting differential protein abundance in mitochondrial‐enriched samples between HL and control conditions, as well as between FUM‐treated and control plants. The curved lines represent threshold boundaries for statistical significance and fold change, visually identifying differentially expressed proteins (*n* = 3, using Student's t‐tests, with an S_0_ value of 0.5 applied to stabilize the test statistic, FDR threshold = 0.05) each biological replicate was derived from an individual mitochondria enrichment from separate pools of biological material. (D) Venn diagram comparing proteins with increased abundance identified in treatments stated in (C). (E) Cellular component gene ontology (GO) term enrichment of 248 proteins with significantly increased abundance after HL exposure. (F) Table of proteins significantly associated with the mitochondrial‐ER interface in Arabidopsis or with homologs in other model organisms linked to mitochondrial‐ER interactions or mitochondrial morphology regulation [114, 115].

To investigate the molecular basis of mitochondrial responses to HL in epidermal tissues, we performed a proteomic analysis using transgenic *pATML1:3xHA‐sGFP‐TOM5* plants, which express GFP‐tagged TOM5, under an epidermis‐specific promoter. Confocal imaging supported the tissue specificity of *pATML1:GFP‐TOM5* expression, as GFP fluorescence was exclusively observed in epidermal tissues, whereas MitoTracker Red staining revealed comparable mitochondrial densities in both epidermal and mesophyll cells (Figure 6A). Western blot analysis of GFP‐TOM5 in total protein from isolated epidermal and mesophyll cells confirmed the selective expression of *pATML1:GFP‐TOM5* in epidermal cells (Figure 6B upper panel; Figure S6A). These findings validate *pATML1* as a robust epidermal‐specific promoter and confirm its effectiveness in labeling mitochondria within epidermal cells.

We enriched epidermal mitochondria from *pATML1:GFP‐TOM5* plants using magnetic beads conjugated to HA‐binding antibodies. Enrichment was validated by immunoblotting for mitochondrial markers IDH and COX2. Minimal chloroplast contamination was confirmed by the highly reduced RbcL signal, while CAT3 and BIP3 served as peroxisomal and ER contamination controls (Figure 6B lower panel, Figure S5B). Although some ER and peroxisomal contamination remained, likely due to their close association with mitochondria, these results confirm successful enrichment of epidermal mitochondria for downstream proteomic analysis.

To assess mitochondrial proteome changes in response to HL, we conducted label‐free quantitative proteomics on enriched mitochondrial samples from HL‐treated, FUM‐treated, mock‐treated, and untreated control plants. As FUM did not induce mitochondrial elongation, it served as an additional negative control. Of the 1,771 protein groups detected, SUBA5 localization analysis showed a ∼5‐fold enrichment of mitochondrial proteins compared to the *Arabidopsis* reference proteome, further confirming successful mitochondrial enrichment (Figure S5C). Volcano plot analysis revealed significant changes in protein abundance across treatments (Figure 6C; Table S2), identifying 248 proteins uniquely upregulated under HL (Figure 6D). Further supporting the reproducibility of our enrichment and proteomics experiments, only 24 proteins were significantly upregulated in the mock‐treated samples compared to the controls, with just 4 exhibiting more than a twofold increase in abundance (Figure S6D). Cellular compartment gene ontology (GO) term enrichment analysis of the 248 proteins significantly upregulated exclusively in HL‐treated samples revealed an enrichment in cellular components associated with mitochondrial membranes, particularly the outer membrane, as well as associated mitochondrial protein complex structures (Figure 6E). Enrichment analysis of biological process GO terms and KEGG representation of TCA cycle and branched chain amino acid degradation pathways, further demonstrates a strong enrichment in respiratory activities and amino acid catabolic process which feed into the TCA cycle in times of stress (Figure S7A–C) [60, 61, 62]. These findings suggest that HL triggers a distinct mitochondrial proteomic signature, consistent with the hypothesis that increased mitochondrial fusion promotes the adaptation of mitochondria activities to increase respiratory activity.

Among the membrane‐associated proteins enriched under HL treatment (Figure 6E), we identified several with known roles at the MERCs or established functions in regulating mitochondrial morphology in other systems, including MIRO1, MIRO2, TRB1, and Sar1b (Figure 6F). MIRO proteins are key regulators of mitochondrial transport and dynamics, mediating interactions with the cytoskeleton and ER [63, 64, 65, 66, 67]. The *Arabidopsis* MIRO2 homolog influenced mitochondrial size and ER tethering when transiently expressed in tobacco [68]. Sar1b, a small GTPase involved in ER‐to‐Golgi trafficking, has been implicated in modulating organelle contacts, including MERCs, in other model systems [69, 70]. TRB1, a TraB family protein, functions as a mitochondrial‐localized tether at MERCs and is essential for mitochondrial morphology and energy metabolism in *Arabidopsis* [71].

The enrichment of these proteins under HL suggests that MERCs may play a critical role in coordinating mitochondrial adaptation to high light stress.

### 2.6 High Light‐Induced ER Substructural Reorganization Is Chloroplast‐Dependent

**FIGURE 7:**
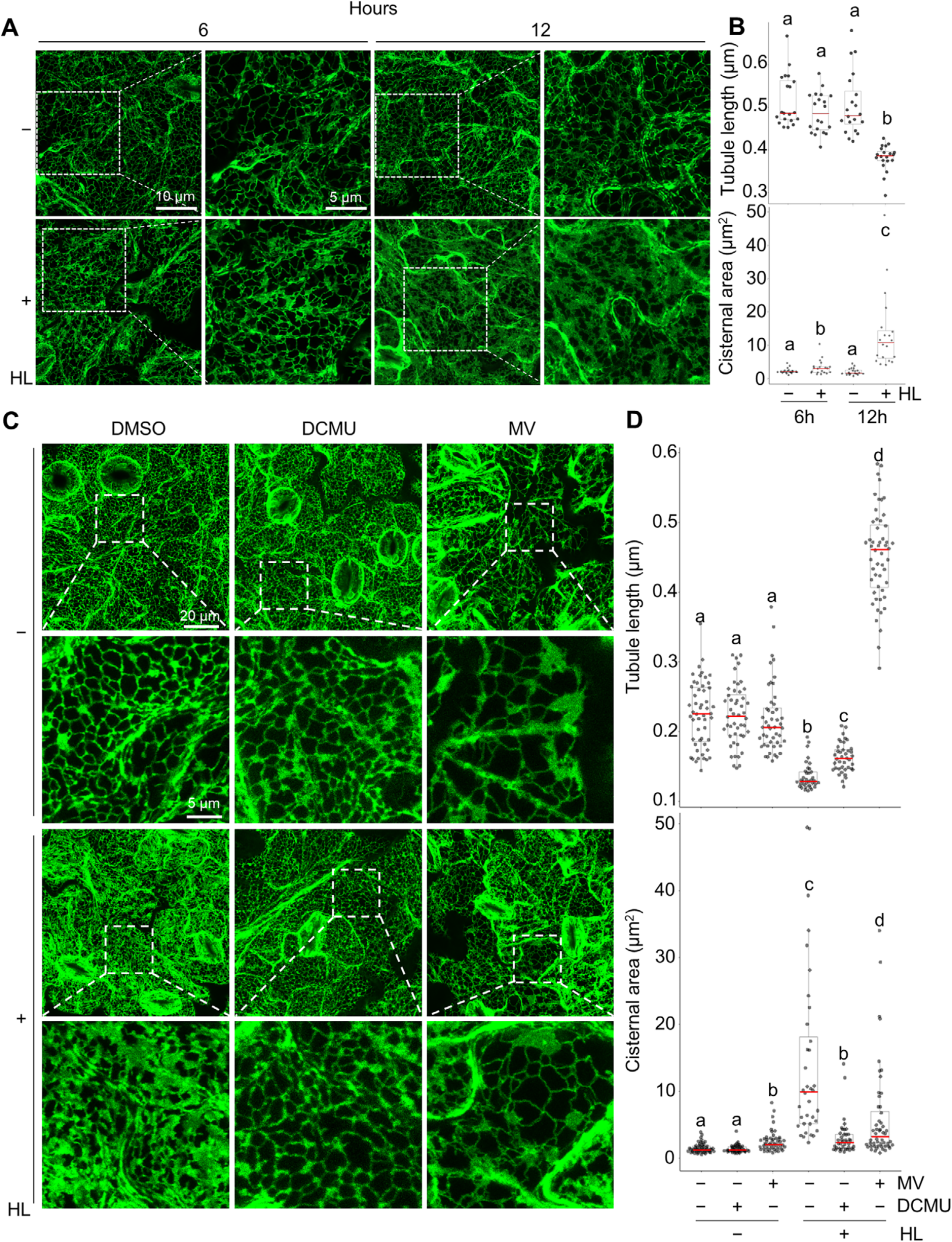
Functional Chloroplasts Contribute to High Light‐Triggered ER Cisternal Expansion. (A,B) Representative images and quantification of ER morphological traits under standard light (HL−) and after 6 and 12 h of high light (HL+) exposure, magnified insets are shown outlined by dotted white lines. Scale bars: 5 or 10 µm, as indicated. (B) Quantification of average ER tubule length and cisternal area in individual cells following 6 and 12 h of high light exposure. Letters denote statistically distinct groups: two‐way ANOVA; Tukey's post‐hoc test; *n* = 20 cells; α = 0.05. (C,D) Representative images of ER morphology in plants treated with DMSO (vehicle control) or 10 µM PSI and PSII inhibitors (DCMU, MV) under HL+ or HL− conditions for 12 h. Magnified insets (outlined by dotted white lines) highlight structural changes. Scale bars: 5–20 µm, as indicated. (D) Quantification of average ER tubule length and cisternal area individual cells under HL+ or HL− conditions following treatment with DMSO, 10 µM DCMU, or 10 µM MV. Letters denote statistically distinct groups: two‐way type III wANOVA; Tukey's post‐hoc test; *n* = 36–52 cells; α = 0.05.

Considering the extensive physical and functional interactions between the ER and mitochondria [31], we investigated whether and how HL could influence ER structural organization. To this end, we used the AnalyzER [45] program to quantify changes in ER substructures, including cisternae, tubules and polygons in cortical ER (Figure S8A,B), by imaging an ER‐YFP marker line [72], under standard light and after 6‐, 12 h of HL exposure. These analyses revealed that HL induces ER cisternal expansion, with concomitant decreases in tubule length and polygonal area becoming prominent by 12 h (Figure 7A,B; Figure S9A), supporting a dynamic restructuring of ER morphology in response to HL.

To determine whether these HL‐induced ER changes depend on photosynthetic electron flow, we treated plants with the PSI and PSII inhibitors MV and DCMU, respectively. ER morphology analysis of plants treated with DMSO or photosynthetic inhibitors under HL or control conditions for 12 h demonstrated that HL‐induced cisternal expansion was abolished in DCMU‐treated plants and strongly reduced MV‐treated plants (Figure 7C,D), demonstrating that chloroplast‐driven photosynthetic activity is required for HL‐induced ER remodeling of cisternae. Interestingly, however, while tubular shortening and reductions in polygonal area were partially recovered in HL+DCMU‐treated plants compared to HL+DMSO treated plants, HL+MV‐treated plants showed increases in tubular length and polygonal area such that they were longer and larger, respectively, than SL controls (Figure 7C,D; Figure S9B), suggesting that, under HL conditions, PSII activity specifically regulates ER tubule‐polygon dynamics, while overall ER restructuring of cisternae depends on active photosynthetic electron flow.

Collectively, our findings support the notion that functional chloroplasts regulate ER substructural organization under HL, specifically by promoting ER cisternal expansion and tubule retraction. These processes are dependent on photosynthetic electron flow, establishing a regulatory link between chloroplast metabolism and ER dynamics. The differential effects of PSI inhibition on ER tubule dynamics suggest a distinct regulatory role for PSII‐driven signaling in overall ER remodeling.

### 2.7 MIRO1 Overexpression Modulates Mitochondrial and ER Morphodynamics

To investigate the role of MIRO1 and other potential candidates (Figure 6F) involved in mitochondria morphodynamics, we transiently overexpressed MIRO1, TRB1 and Sar1B in tobacco leaves using a dual expression construct that co‐expressed a mitochondrial matrix localized mito‐mCherry [72], and N‐terminal GFP fusions of our proteins of interest. MIRO2 overexpression in tobacco has been demonstrated to alter mitochondria morphology [68] and was therefore not tested in this study. MIRO1 overexpression led to significant mitochondrial elongation, consistent with previous studies demonstrating MIRO1's role in mitochondrial dynamics [73, 74], whereas control (empty vector; EV), Sar1b, and TRB1‐overexpressing plants did not exhibit such changes (Figure S10A,B). To rule out differences in the accumulation of GFP‐fusion proteins at mitochondria as a confounding factor, we assessed the mCherry/GFP fluorescence intensity ratio in transient expression assays. Results confirmed comparable fluorescent protein expression levels across candidate constructs (Figure S10C).

**FIGURE 8:**
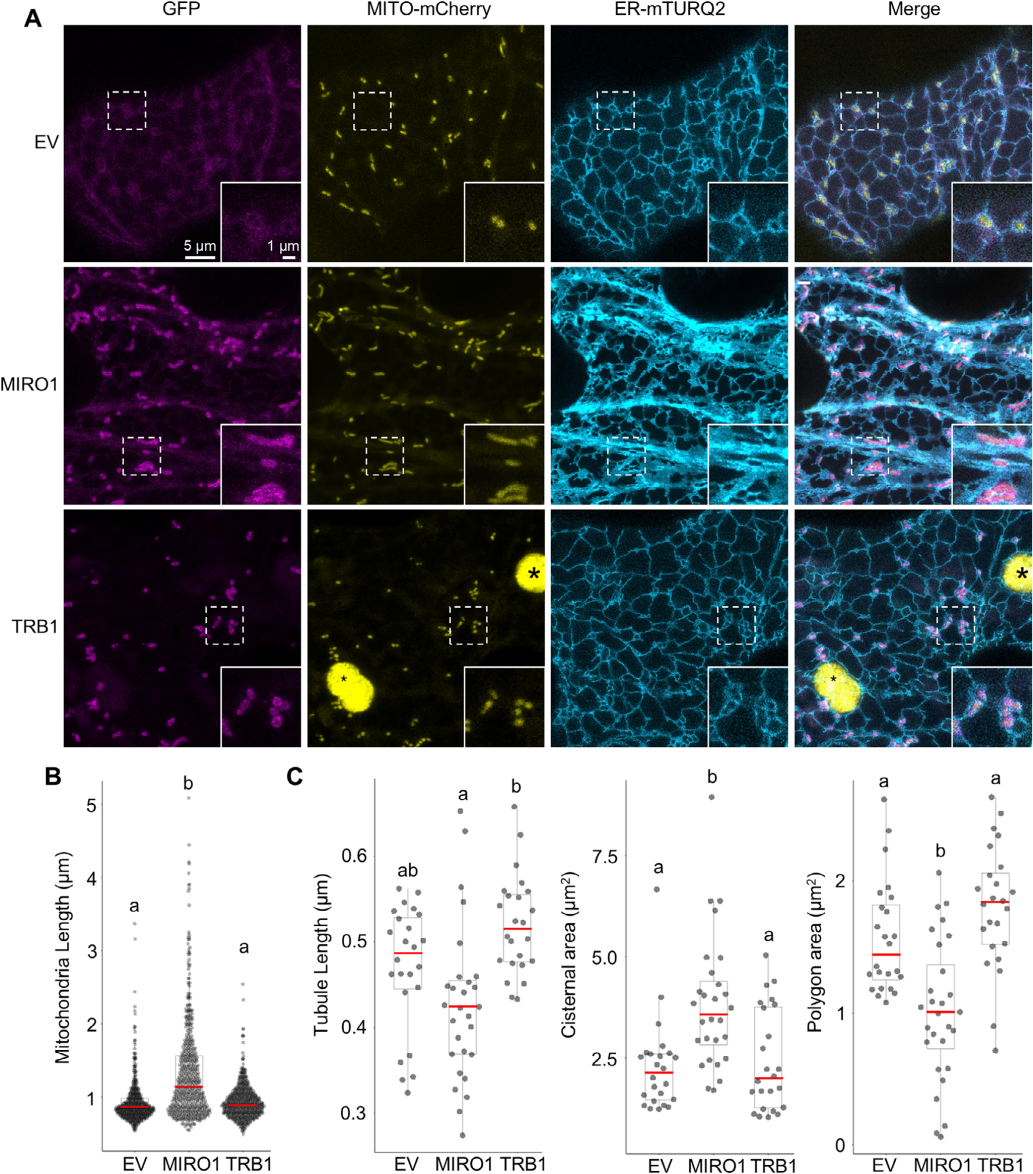
MIRO1 Overexpression Drives Mitochondrial Elongation and ER Cisternal Expansion. (A) Representative fluorescence images of tobacco leaves transiently co‐expressing a dual overexpression construct containing a Mito‐mCherry and either a cytosolic eGFP (i.e. empty vector; EV), N‐terminal eGFP‐MIRO1 or eGFP‐TRB1, and a second construct expressing ER‐mTURQ2‐HDEL. Magnified insets, outlined by dotted white lines, are shown in the lower right corner of each image. Scale bars: 1 or 5 µm as indicated; chlorophyll autofluorescence indicated by *. (B,C) Quantification of mitochondrial length (B) and ER substructure morphologies (C), including tubule length, cisternal area, and polygonal area in tobacco leaves overexpressing EV, eGFP‐MIRO1, and eGFP‐TRB1. Letters denote statistically distinct groups: (B) one‐way type III wANOVA; Tukey's post‐hoc test; *n* = 480–560 mitochondria; (C) one‐way type III ANOVA; Tukey's post‐hoc test; *n* = 24–28 cells; α = 0.05.

We then co‐expressed the dual reporter constructs described above with an ER lumen localized mTurqoise2 (ER‐mTURQ2), using EV and TRB1 as negative controls. In addition to inducing mitochondrial elongation, MIRO1 overexpression caused marked structural changes in the ER, including an expansion of the cisternal network (Figure 8A–C). These alterations mirror the ER reorganization observed in Arabidopsis following HL treatment (Figure 7), suggesting that MIRO1 may mediate functional interactions between mitochondria and the ER. This is reminiscent of findings in mammalian systems, where MIRO1 plays a role in ER‐mitochondria tethering and calcium exchange [67, 75]. Collectively, these results demonstrate that MIRO1 overexpression promotes mitochondrial elongation and modulates ER structure, potentially enhancing inter‐organelle communication.

### 2.8 Acetylation and Calcium‐Binding Ability of MIRO1 Regulate Mitochondrial Dynamics and Motility

To investigate the molecular mechanisms underlying MIRO1 function, we examined its structural conservation and potential post‐translational modifications. In the absence of fully developed crystal structures, we exploited AlphaFold‐predicted models to compare *Arabidopsis* and human MIRO1 which revealed high structural similarity in the GTPase and EF‐hand calcium‐binding domains (GTPase1 + EF1: 99%; GTPase2 + EF2: 96%), despite relatively low sequence identity (43% and 30%, respectively; Figure S11A–D). This strong structural conservation supports the functional conservation of MIRO1 across species, consistent with previous studies of MIRO1 homologs [63, 66, 73, 76].

Given the established role of MIRO1 post‐translational modifications (PTMs) in regulating mitochondrial dynamics in mammals, such as K105 acetylation essential for mitochondrial transport [77], and S66 phosphorylation by Polo kinase modulating ER‐mitochondria contact sites [78], we examined putative PTMs of Arabidopsis MIRO1 using publicly available proteomics datasets (Figure S11B,C) [79, 80]. We also generated MIRO1 variants targeting conserved functional domains, including GTPase activity and calcium binding, based on known mammalian MIRO1 mutations [81].

While the dominant‐negative (DN) GDP‐bound GTPase1 mutation (S31N) is conserved, the mammalian constitutively active (CA) mutation (P14V) was not. Through structural alignment with the known CA variant of the Arabidopsis small GTPase, ROP2 (G12V) [82], we identified G27 in *Arabidopsis* MIRO1 as structurally analogous and generated the G27V CA mutant.

**FIGURE 9:**
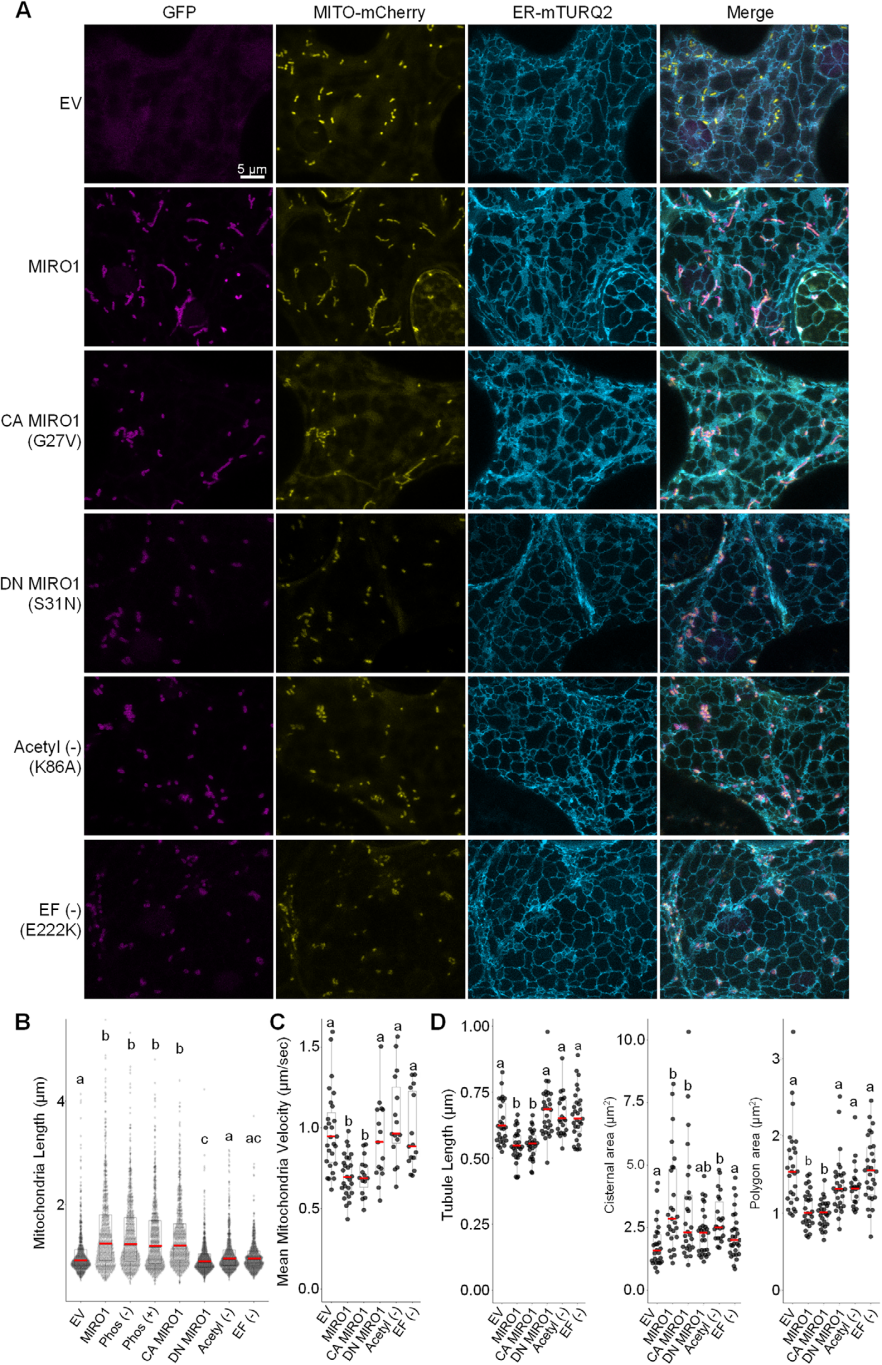
Acetylation and Calcium‐Binding Ability of MIRO1 Regulate Mitochondrial Morphodynamics and Motility. (A) Representative confocal images in transiently transformed tobacco leaves expressing the empty vector (EV) control, wild‐type MIRO1, and MIRO1 mutants, including constitutively active (CA MIRO1; G27V), dominant‐negative (DN MIRO1; S31N), putative acetylation‐deficient (Acetyl(‐); K86A), and calcium‐binding‐deficient (EF(‐); E222K) variants and co‐transformed with a second construct expressing ER‐mTURQ2‐HDEL. Scale bar = 5 µm. (B) Quantification of mitochondrial length across different dual reporter MIRO1 constructs including EV and MIRO1 control, the phospho‐deficient (Phos(‐); S14A), phospho‐mimetic (Phos(+); S14E), and CA MIRO1, DN MIRO1, Acetyl(‐), or EF(‐) mutants, representative images for this experiment found in Figure S12B. Letters denote statistically distinct groups: one‐way wANOVA; Tukey's post‐hoc test; *n* = 640 mitochondria; α = 0.05. (C) Analysis of mitochondrial velocity, revealing a reduction in mitochondrial movement in tobacco transiently transformed with EV, wild‐type MIRO1 and CA MIRO1, DN MIRO1, Acetyl(‐), or EF(‐) mutants. Letters denote statistically distinct groups: one‐way type III wANOVA; Tukey's post‐hoc test; *n* = 28 recordings (EV and MIRO1), 16 recordings (MIRO1 variants); collective results from two separate experiments, data from EV and MIRO1 controls are pooled; α = 0.05. (D) Quantification of ER substructure morphologies from the experiment in (A), including tubule length, cisternal area, and polygonal area in tobacco leaves transiently overexpressing EV, MIRO1 and the indicated MIRO1 mutant constructs. Letters denote significance: one‐way type III ANOVA (tubule and polygonal area) or one‐way type III wANOVA (cisternae); Tukey's post‐hoc test; *n* = 22–29 cells; α = 0.05.

Expression levels of all MIRO1 variants were comparable (Figure S12A), allowing us to assess their effects on mitochondrial morphology. Overexpression of wild‐type MIRO1, the phosphorylation mimic (S14E), phosphorylation‐deficient (S14A), and CA‐MIRO1 (G27V) induced significant mitochondrial elongation. In contrast, the DN (S31N), acetylation‐deficient (K86A), and calcium‐binding‐deficient (E222K) mutants failed to do so (Figure 9A,B; Figure S12B).

Mitochondrial motility analysis revealed an inverse relationship between elongation and movement: wild‐type and CA‐MIRO1 reduced motility, while the DN, K86A, and E222K mutants maintained velocities comparable to EV controls (Figure 9C). Co‐expression of these MIRO1 variants with the ER marker ER‐mTURQ2 showed that CA‐MIRO1 preserved the ER phenotype observed with MIRO1 overexpression, whereas DN, K86A, and E222K mutants resulted in ER morphology similar to controls (Figure 9A,D). These results suggest a crucial role for calcium binding and putative acetylation in MIRO1‐mediated mitochondrial dynamics, in agreement with prior reports implicating MIRO1 in cytoskeleton‐mediated mitochondrial positioning and movement [81, 83, 84].

These findings suggest that MIRO1 actions at the mitochondrial outer membrane mediate mitochondria motility, mitochondrial elongation, and ER morphology, reinforcing the regulatory importance of MIRO1 post‐translational and regulatory modifications in coordinating mitochondrial morphodynamics and mobility, consistent with previous studies linking MIRO1 to mitochondrial transport and fusion‐fission balance [66, 85].

## 3 Discussion

Chloroplasts serve as a central hub for cellular metabolism, integrating energy production, biosynthesis, and redox homeostasis. Their function is finely regulated by both intrinsic genetic programs and extrinsic environmental cues. Perturbations in chloroplast function, whether through genetically induced disruptions in the MEP‐pathway or environmental challenges such as HL stress, trigger a cascade of metabolic and structural changes that extend beyond the plastid itself. One striking consequence of such perturbations is the reconfiguration of mitochondrial morphology, particularly elongation, which reflects a coordinated interorganellar response to maintain cellular homeostasis.

### 3.1 Cell‐Type‐Specific Mitochondrial Elongation in Response to High Light

Our findings reveal a striking cell‐type‐ and surface‐specific response to HL stress: mitochondrial elongation occurs predominantly in epidermal pavement cells on both the adaxial and abaxial leaf surfaces, but not in mesophyll cells, where most photosynthesis takes place. The occurrence of elongation in both epidermal layers suggests that this response is not simply driven by differential light exposure but rather reflects an intrinsic property of epidermal cells. Epidermal mitochondria may be more responsive to systemic or chloroplast‐derived oxidative signals transmitted across tissue layers, or they may possess a lower antioxidant buffering capacity than mesophyll cells. In contrast, mesophyll mitochondria, situated in cells specialized for photosynthesis, may maintain a more robust redox homeostasis that prevents morphological remodeling under HL stress. Collectively, these observations point to an epidermis‐specific adaptation to HL‐induced oxidative signaling, highlighting spatial compartmentalization of redox communication and organellar dynamics within the leaf.

Mitochondrial elongation has been proposed as an adaptive mechanism to counteract oxidative stress by improving ATP production efficiency and facilitating mitochondrial content mixing through fusion [19, 21]. This response may function as a protective strategy to sustain energy production while minimizing damage from excess ROS generated during HL exposure. Additionally, it may help meet the elevated ATP demands of epidermal mitochondria under HL stress, which are required to support critical cellular functions such as cuticle biosynthesis, defense activation, and stress signaling [86, 87]. Given that mitochondrial elongation increases cristae surface area and enhances respiratory efficiency, this morphological adaptation may serve to meet these elevated energy requirements.

This epidermis‐specific mitochondrial elongation aligns with previous observations that epidermal plastids exhibit distinct structural features and specialized functions compared to mesophyll chloroplasts. These “sensory plastids” possess a unique proteome enriched in stress‐associated proteins, highlighting their role in environmental sensing and adaptive responses [88]. Together, these unique mitochondrial and chloroplast features underscore the epidermal tissue's role as a dynamic interface between plants and their environment, orchestrating early stress perception and adaptive responses, such as the regulation of stomatal behavior, cuticle biosynthesis, and activation of stress‐responsive gene networks to ensure whole‐plant survival.

Our findings suggest that tissues directly exposed to environmental stressors have evolved specialized organelle functions, serving as the first line of defense against abiotic challenges. This highlights the necessity of cell‐type‐specific adaptations in organelle function and provides a mechanistic framework for how interorganellar communication optimizes metabolic restructuring, enhancing plant resilience to environmental stress.

### 3.2 Chloroplast‐Driven Regulation of Mitochondrial Elongation

Our pharmacological studies demonstrated that inhibition of PSI and PSII abolished HL‐induced mitochondrial elongation, whereas inhibition of the mitochondrial ETC had no suppressive effect. This suggests that the signal regulating mitochondrial elongation originates from the chloroplast rather than an internal response to altered mitochondrial metabolic activity.

A likely candidate for this regulation is chloroplast‐derived ROS. HL exposure accelerates electron transport in PSII, leading to the overproduction of superoxide and hydrogen peroxide within 1 h after exposure [1, 89]. Hydrogen peroxide, in turn, act as 1) an intracellular retrograde signal to regulate nuclear gene expression and cellular stress responses [2, 51], and 2) an intercellular signal propagating between cells through plasmodesmata, while additionally initiating extracellular respiratory burst oxidase, and calcium waves to signal systemic tissues [90, 91]. The involvement of hydrogen peroxide signaling is supported by reports that have demonstrated PSII inhibition by DCMU also abrogated cellular accumulation of hydrogen peroxide after HL treatment [89]. Consistent with this, exogenous H_2_O_2_ application mimicked the HL‐induced mitochondrial elongation, whereas antioxidant pretreatments failed to block this response, suggesting that localized or transient H_2_O_2_ accumulation, rather than bulk redox status, is critical for initiating the morphological transition. Moreover, chelating intracellular Ca^2^^+^ with BAPTA‐AM abolished mitochondrial elongation induced by both HL and H_2_O_2_, establishing Ca^2^^+^ signaling as an essential downstream component of H_2_O_2_‐mediated mitochondrial remodeling.

Mitochondria are highly sensitive to ROS fluctuations, and previous studies in mammalian models have shown that mitochondrial elongation is a possible response to oxidative stress, enabling more efficient ATP production and reducing ROS damage through hyper‐fusion [22, 23, 92]. However, a redox sensitive plant specific effector of these processes remains to be identified as the MFN proteins which mediate this process in metazoans are not conserved in plants [44].

Alternatively given the calcium‐dependency of MIRO1‐mediated mitochondrial elongation (Figure 9), it may be possible that increases in cytosolic calcium concentrations either as a consequence of cellular ROS buildup [90, 91], or through a combinatorial ROS‐Ca^2^^+^ signaling mechanism promote mitochondria fusion following HL exposure. Supporting this notion, recent work has demonstrated that photo‐stimulation of the chloroplast‐ER junction triggers transient spikes in cytosolic Ca^2^^+^ levels, and remodels cortical ER morphology [93, 94], suggesting that HL‐induced mitochondrial elongation may be part of a coordinated, multi‐organelle response integrating chloroplast, ER, and mitochondrial signaling.

### 3.3 Early Suppression of Mitochondrial Motility and Structural Coupling Precede Elongation

Our time‐course analysis revealed that mitochondrial motility is reduced within one hour of HL exposure, preceding elongation and fusion. This sequence of events suggests that reduced mitochondrial movement is an early response to stress, likely to serve to stabilize mitochondrial positioning, potentially priming organelles for network integration.

One plausible mechanism involves cytoskeletal regulation: long‐range mitochondrial transport is primarily mediated by the coupling of mitochondria to microtubule motors (kinesins and dynein), whereas the actin cytoskeleton and myosin motors mediate short‐range movement and anchoring [95, 96, 97]. HL‐induced ROS signaling may trigger actin cytoskeleton remodeling, leading to tighter mitochondrial anchoring to facilitate subsequent fusion [95, 98, 99]. This aligns with recent observations that both mitochondria‐associated and ER‐associated actin are required for effective execution of mitochondrial fusion [29].

Calcium signaling may also contribute to reduced mitochondria movement. MIRO1 is known to mediate calcium‐dependent mitochondrial arrest in neurons [76]. Our proteomic analysis revealed an increased MIRO1 abundance in epidermal mitochondria under HL conditions. Functional analysis using an EF1 domain mutant, disrupting MIRO1's calcium‐binding capacity, demonstrated that this calcium‐sensing function is required for HL‐induced motility suppression. These findings suggest that elevated cytosolic Ca^2^^+^ levels under HL activate MIRO1, leading to a reduced mitochondrial trafficking and priming mitochondria for structural remodeling.

Consistent with this model, TEM and electron tomography revealed that HL promotes the formation of membrane contact sites between adjacent mitochondria, characterized by parallel outer membrane alignment and occasional tether‐like connections. These structural features, absent under standard light, indicate that close physical proximity is an early and active component of the mitochondrial remodeling process rather than a mere byproduct of fusion. Together, these results suggest that HL stress initiates a coordinated response involving reduced motility, inter‐mitochondrial tethering, and membrane reorganization, establishing the structural conditions necessary for fusion‐driven elongation.

### 3.4 Chloroplast‐Dependent ER Remodeling under High Light

Beyond the chloroplast's role in mitochondrial adaptation, our findings reveal that HL stress also induces extensive remodeling of the endoplasmic reticulum ER, a central organelle in lipid biosynthesis, calcium signaling, and protein homeostasis [100, 101]. The ER dynamically restructures in response to metabolic demands and environmental cues, often undergoing tubule shortening and cisternal expansion to facilitate membrane trafficking, protein processing, and inter‐organelle communication [102, 103, 104]. These morphological adaptations underscore the ER's integral role in cellular stress responses.

We observed pronounced ER remodeling, marked by shortened tubules and expanded cisternae. This restructuring was abolished when photosynthetic electron transport was inhibited, indicating a chloroplast‐dependent mechanism. Notably, MIRO proteins were enriched in HL‐treated mitochondria, and MIRO1 overexpression alone was sufficient to reproduce the HL‐induced ER phenotype. This suggests that ER remodeling may arise as a secondary consequence of altered ER‐mitochondrial interactions supporting mitochondrial function under HL stress.

In mammalian systems, MERCs regulated calcium exchange is essential for mitochondrial dynamics [105, 106]. MIRO proteins promote MERC formation and facilitate calcium delivery to mitochondria [107], and further directly regulates the IP3R‐VDAC (Inositol 1,4,5‐trisphosphate receptors‐ voltage‐dependent anion channel) complex responsible for this calcium transfer [78]. While VDACs are conserved in higher plants, IP3Rs are not [108]. Nevertheless, ER calcium release has been observed in plants in response to specific stimuli, although the channels responsible for these releases remain unidentified [109, 110, 111]. We propose that MIRO1 may facilitate mitochondrial calcium uptake in plants through mechanisms that require ER morphological adaptation to meet mitochondrial calcium demand. Supporting this hypothesis, disruption of ER tubule structure, such as through loss of the reticulon protein RTN4, impairs ER calcium uptake and alters internal calcium transport by modifying tubule architecture [112]. Therefore, HL‐induced ER sheet expansion and tubule retraction may reduce ER calcium sequestration, thereby increasing calcium availability to mitochondria. Additionally, changes in tubule width are known to regulate bulk calcium flow within the ER [104], further suggesting that ER remodeling under HL may optimize inter‐organelle calcium exchange in support of mitochondrial function.

Alternatively, ER restructuring may be a consequence of increased lipid exchange between the ER and chloroplasts. As the ER supplies lipids for thylakoid membrane biogenesis, accelerated thylakoid turnover under HL may necessitate rapid ER restructuring to accommodate elevated lipid exchange [113]. The involvement of MIRO1 and MIRO2 in mitochondrial‐ER tethering supports a model in which HL‐induced remodeling of both organelles is coordinated through inter‐organelle crosstalk.

In summary, HL elicits a sequential mitochondrial response in epidermal cells, beginning with reduced motility, followed by the formation of membrane contact sites between adjacent mitochondria, and culminating in elongation via increased fusion. This response is dependent on chloroplast activity but occurs independently of mitochondrial electron transport or metabolic flux. In parallel, HL enhances ER remodeling and enriches proteins at the mitochondria‐ER interface, indicating elevated inter‐organelle communication under stress. These findings highlight a tightly coordinated network involving chloroplasts, mitochondria, and the ER that enables spatially resolved adaptation to environmental challenges. Future studies should focus on elucidating the signaling mechanisms, particularly the roles of ROS and calcium, that orchestrate this integrated organellar response.

## 4 Methods

### 4.1 Experimental Model and Subject Details

All *Arabidopsis thaliana* wild‐type and transgenic lines plants used in this study were in the Col‐0 ecotype. The *HDSi* [116], *3x‐HA‐sGFP‐TOM5* [56], *SHMT‐roGFP2‐Grx1* [58], and *ER‐YFP‐HDEL* [72] (*er‐yk*) lines have been described previously. *HDRi*, *DSXi*, and *pATML1:GFP‐TOM5* lines were generated in this study, and only single‐insert transformants homozygous for the respective transgene were used. Lines expressing 3x‐HA‐sGFP‐TOM5 in the *HDSi*, *HDRi*, and *DXSi* backgrounds were generated by crossing *3x‐HA‐sGFP‐TOM5* with each RNAi line. All *Nicotiana benthamiana* plants used in this study were wild‐type (WT).

For all Arabidopsis experiments, seeds were surface sterilized using a 50% bleach solution, rinsed 5 times with double distilled sterile water (hereafter sterile water) and then sown on freshly prepared on half strength Murashige and Skoog basal medium (2.16 g L^−1^; PlantMedia, Dublin, OH, USA) supplemented with MES (1 g L^−1^, 2‐(N‐morpholino) ethanesulfonic acid) and Phytoagar (8 g L^−1^; PlantMedia, Dublin, OH, USA) at pH 5.7 (adjusted with 1 M potassium hydroxide). Plate edges were wrapped in 1‐inch surgical tape (Millipore, Burlington, MA, USA), and then sown seeds were cold‐stratified at 4°C for 2–3 days. After stratification, plates were transferred to a Percival growth chamber maintained at 22°C under long day conditions (16 h light/8 h dark), and 100 ± 10 µmolm^−2^ s^−1^ light intensity. For the experiment in Figure 1J examining mitochondria morphology in root tissues, Phytoagar was replaced with plant tissue culture agar (10 g L^−1^; Neogen, Lansing, MI, USA) and plates were incubated vertically to allow for root growth along the surface of the media instead of penetrating the media.

Tobacco seeds were germinated directly on wetted Sunshine Mix #5 Propagation Mix (SunGro, Agawam, MA, USA) in temperature‐controlled growth room at 22°C under long day conditions (16 h light/8 h dark), and 100 ± 10 µmol m^−2^ s^−1^ light intensity. Plants used for transient transformation and heterologous expression were between 2.5‐4 weeks of age. Photosynthetically active radiation, and spectrum information for all light conditions were collected using a VBR‐100 (400‐700 nm) and VBR‐300 for UVA (365‐400 nm) and far‐red light (730 nM; VABIRA, Zhongshan City, Guangdong, China).

### 4.2 Methods Details

#### 4.2.1 Plasmid Construction

Whole or partial coding sequences for all genes used in this study were amplified from cDNA derived from WT Arabidopsis unless indicated. Dexamethasone inducible RNAi constructs (*DXSi* and *HDRi*) were generated by amplifying 100–200 bp regions close to the n‐terminus of each cDNA sequence using primers (1‐4) (See Table S3 for all primers) and subcloned into the pDONR207 entry vector by BP Clonase II reaction (ThermoFisher Scientific, Waltham, MA, USA), and then sequence verified by Sanger sequencing before being recombined into the RNA hairpin producing pOpoff2 vector [117], by LR Clonase II reaction (ThermoFisher Scientific, Waltham, MA, USA). The pATML1:GFP‐TOM5 construct was generated in the pCambia1300 vector by amplification of a 3415 bp fragment of the ATML1 promoter from WT Arabidopsis genomic DNA using primers (5‐6), and amplification of 3x‐HA‐sGFP‐TOM5 gene using primers (7‐8), from the genomic DNA of the respective *3x‐HA‐sGFP‐TOM5* plant line [56]. The pCambia1300 vector was digested by HindIII and SalI restriction enzymes (RE; NEB, Ipswitch, MA, USA) and then assembled with the pATML1 and 3x‐HA‐sGFP‐TOM5 DNA fragments via NEB Hifi Assembly reaction (NEB, Ipswitch, MA, USA), then Sanger sequence verified.

The dual expression construct, pMIT, was generated by amplifying the eGFP‐Gateway cassette from the pB7WGF2 binary vector [118] using primers (9‐10) and inserted into the pCAMBIA1300‐UBQpro‐MCS vector [119] at the KpnI (NEB, Ipswitch, MA, USA) RE site via NEB Hifi Assembly reaction to generate pCAMBIA1300‐UBQpro‐GFP‐GW. Then the fragment containing the pUBQpro‐GFP‐Gateway‐HSP18.2 terminator from pCAMBIA1300‐UBQpro‐GFP‐GW was amplified using primers (11‐12) and inserted into the mt‐rb binary vector [72] at the PvuI (NEB, Ipswitch, MA, USA) RE site via NEB Hifi Assembly reaction and Sanger sequence verified. This generated the pMIT vector which expresses a 35S:COX4‐mCherry [72] (localized to the mitochondrial matrix) and a ubiquitin promoter driven N‐terminal eGFP fusion.

To generate N‐terminal GFP fusion constructs of candidate proteins, the sequence verified MIRO1 cDNA in the pENTR‐SD‐TOPO vector was obtained from Arabidopsis Biological Resource Center [120]. The TRB1 and Sar1b coding sequences (CDS) were amplified from cDNA using a two‐step PCR process, with an initial amplification using gene specific primers containing small overhangs (primers 13–16), which were extended to full attb1/2 adapters with a second PCR using universal primers (primers 17–18). TRB1 and Sar1b CDS were subcloned into pDONR207 via BP Clonase II reaction. Generation of MIRO1 mutant variants was performed with the Q5 Site Directed Mutagenesis Kit (NEB, Ipswitch, MA, USA), using primers 19–30, on the pENTR‐MIRO1 entry vector. Mutations were verified by Sanger sequencing. Candidates and MIRO1 variants were then recombined into the pMIT dual expression vector via LR Clonase II reaction.

To generate the pCAMBIA1300‐UBQpro‐ER‐mTURQ2 construct a DNA fragment was synthesized (TwistBio, South San Francisco, CA, USA) containing the Arabidopsis Calreticulin 1 (CRT1) ER signal sequence N‐terminally fused to mTURQ2 [121] which was codon optimized for Arabidopsis expression, and a C‐terminal HDEL retention signal (full sequence provided in Table S3, #31). The fragment had DNA synthesis adapters removed by EcoRV (NEB, Ipswitch, MA, USA) digestion and then the fragment was inserted into the pCAMBIA1300‐UBQpro‐MCS [119] vector at the KpnI site via NEB Hifi Assembly reaction.

#### 4.2.2 Plant Transformation

To generate Arabidopsis stable transformants for the HDRi, DXSi, and pATML1:TOM5 GFP constructs, binary plant vectors were transformed into *Agrobacterium tumefaciens* strain GV3101 and flowering Col‐0 plants were floral dipped, then selected for single‐insertion transformants by screening on sterile media containing kanamycin (50 µg mL^−1^).

Transient transformation of pMIT and ER‐mTURQ2 constructs in *Nicotiana* was performed after binary vectors were transformed into GV3101 [122]. Individual colonies were inoculated into 10 mL Luria‐Bertani (LB) broth containing the appropriate antibiotics, kanamycin (50 µg mL^−1^), gentamycin (30 µg mL^−1^), and rifampicin (20 µg mL^−1^) and grown overnight at 28°C. Culture was collected at OD_600nm_ of less than 1 the following morning, and cells were pelleted by centrifugation at 1000 *g* for 10 min. Cells were washed by resuspension in freshly prepared infiltration buffer (IF) containing 50 mM MES, 5 mg mL^−1^ D‐glucose, 200 µM acetosyringone (all from Sigma–Aldrich, St. Louis, MO, United States), centrifuged again and resuspended in IF buffer. The OD_600nm_ of GV3101 cell suspensions transformed with pMIT‐empty vector (EV), pMIT‐MIRO1 (including MIRO1 variants), and pMIT‐Sar1b, were measured again and further diluted to equivalent OD_600nm_ = 0.005 in IF buffer. pMIT‐TRB1 suspensions were diluted to equivalent OD_600nm_ = 0.0025. When co‐infiltrated with pCAMBIA1300‐UBQpro‐ER‐mTURQ2, resuspended cells were added to equivalent OD_600nm_ = 0.005. Infiltration with very low equivalent OD_600nm_ suspensions was required to prevent confounding effects of extreme mitochondria morphology phenotypes observed at higher OD_600nm_. Agrobacterium suspensions (200–300 µL) were pressure infiltrated into the abaxial side of *Nicotiana* leaves using a needleless 1 mL syringe. For consistency, only the fully expanded first‐ fourth true leaves (first being the initial true leaf that formed after the cotyledons) were infiltrated. Additionally, at least 4 separate infiltrations (1 per leaf) on at least two plants (2 per plant) were performed per construct for each experiment to reduce outlier effects of individual leaves or plants. After infiltration, *Nicotiana* was returned to standard long day growth conditions without humidity covering.

### 4.3 High Light and Dexamethasone and Inhibitor Treatments

For induction of RNAi constructs, 10 mM dexamethasone stock (Sigma–Aldrich, St. Louis, MO, United States) in DMSO was diluted to 30 µM in sterile water. DMSO control and 30 µM dexamethasone was filter sterilized and sprayed on 11‐day old seedlings in culture plates using a small handheld pump‐action sprayer to achieve an even coating over all seedlings. Plates were then re‐wrapped with 1‐inch surgical tape and returned to previously described growth conditions for 3 days.

High light treatment for all experiments was performed in a temperature‐controlled growth room (19°C) using the HL #2 LED panel shown and described in Figure S2E. In accordance with previously published methods to eliminate the confounding effects of heat produced by high light treatment [123], plates (still wrapped with surgical tape) containing seedlings were also subjected to continuous airflow by fan during high light treatment and had their leaf temperature monitored by handheld infrared laser thermometer (Etekcity, Anaheim, CA, USA) to maintain adaxial surface leaf temperatures of 20–22°C. Unless otherwise stated in relevant figure legends or methods, high light treatments for all experiments were performed 11 days after removal from stratification conditions and were treated for 12 h starting at the beginning of the long day.

For treatment with photosystem and mitochondrial ETC inhibitors 10 mM stocks of DCMU, MV (Sigma‐Aldrich, St. Louis, MO, United States) in DMSO were diluted to 5 and 10 µM in sterile water and Antimycin A (ThermoFisher Scientific, Waltham, MA, USA) in DMSO was diluted to 40 µM in sterile water. For fumarate and malate treatments fumaric acid and malic acid (Sigma‐Aldrich, St. Louis, MO, United States) were dissolved at 1 mM in sterile water and neutralized to pH 7 using 1 M potassium hydroxide, then diluted to 10 µM in sterile water. At the beginning of the day, prior to the initiation of high light treatment for relevant experiment, each filter sterilized solution was sprayed onto separate plates of seedlings in the same manner as dexamethasone. Plates were then re‐wrapped with 1‐inch surgical tape and then either returned to standard light growth conditions or subjected to high light treatment for 12 h as indicated. DCMU and MV treatments for 5 and 10 µM were split into two experiments with independent DMSO control groups for each experiment to prevent over extended treatments while imaging many experimental groups.

### 4.4 Hydrogen Peroxide, Calcium Chelator, and Antioxidant Treatments

For experiments testing mitochondrial elongation in response to hydrogen peroxide (H_2_O_2_) (Figure 5A–E), working solutions of 10, 25, 50, and 100 mM H_2_O_2_ (Sigma–Aldrich, St. Louis, MO, USA) were freshly prepared in sterile water. Approximately 1 ± 0.1 mL of each solution was evenly sprayed onto 100 mm × 100 mm square Petri dishes containing 36 eleven‐day‐old seedlings, delivering an estimated 10, 25, 50, and 100 µmol of H_2_O_2_ per plate. After spraying, plates were sealed with 1‐inch surgical tape and returned to standard light growth conditions for the indicated durations.

To enable high‐throughput testing of calcium chelators, antioxidants, and related compounds on mitochondrial elongation under high light, seeds were sown individually in 48‐well plates containing 0.5 mL of half‐strength Murashige and Skoog basal medium per well (as described above) and grown for seven days after germination. Working solutions of BAPTA‐AM (5, 25, 50, and 100 µM; Thermo Fisher Scientific, Waltham, MA, USA), EGTA (50 mM ethyleneglycol‐bis(β‐aminoethyl)‐N,N,Nʹ,Nʹ‐tetraacetic acid; Sigma–Aldrich), ascorbic acid (AsA; 0.25, and 5 mM, neutralized to pH 7 with 1 M KOH; Sigma–Aldrich), and sorbitol (0.25, and 5 mM, osmotic control for AsA; Sigma–Aldrich) were prepared, along with glutathione (GSH, reduced; 0.25 mM; Sigma–Aldrich), N‐acetylcysteine (NAC; 0.25 mM; Sigma–Aldrich), and catalase (100 units mL^−1^, from bovine liver, C‐9322; Sigma–Aldrich). All solutions were prepared in sterile water and filter‐sterilized.

At the beginning of the day, 250 µL of each sterile working solution or mock control was added to individual wells (five seedlings per treatment). Plates were returned to standard light conditions for 2 h. After treatment, solutions were removed by pipette, and excess moisture was eliminated by inverting the plates and gently tapping them over sterile lab wipes. Duplicate plates were then either transferred to high light conditions for 6 h or maintained under standard light. Cotyledons were collected for confocal microscopy analysis.

### 4.5 Confocal Laser Scanning Microscopy

All microscopy experiments, except for those shown in Figure 2E–G, Figure S1E,F, and Movies S1 and S2, were performed on a Zeiss LSM900 inverted confocal microscope (Zeiss, Oberkochen, Germany) equipped with a Plan Acromat 40x/NA1.4 oil objective. Images of the MEP RNAi lines in Figure S1E,F were collected using a Zeiss LSM880 upright confocal microscope (Zeiss, Oberkochen, Germany) with Airyscan equipped with an LD LCI Plan‐Apochromat 40x/1.2 Imm Corr DIC M27 objective for improved resolution in discerning the morphological changes occurring after MEP pathway inhibition. Scans were completed using super resolution mode, and all settings including Airyscan data processing were set to defaults. Excitation by laser line and emission spectra collected for each fluorophore are as follows: eGFP and roGFP2 (ex‐488 nm; em‐ 400–530 nm), YFP (ex‐514 nm; em‐ 400–560 nm), mCherry (ex‐588 nm; em‐ 600–640 nm). For Peredox‐mCherry imaging, two tracks were required to determine fluorescence intensity for the required tSapphire emission spectra (500‐530 nm), the first excited at 405 nm and emission collected at 400–530 nm, the second track was excited at 405 nm and emission collected at 400–500 nm. mCherry signal was collected on the first track.

For experiments examining mitochondria and ER morphology (static images) in Arabidopsis leaf tissues (either true leaf or cotyledons) the following mounting and imaging protocol was followed to reduce possible artifacts due to plants being removed from treatment conditions, and to minimize the time samples were kept under a coverslip, as previous studies have demonstrated an effect of extended time under and coverslip, and hypoxia on mitochondrial dynamics in plant cells [38, 124]. A single plate was removed from treatment conditions, opened, leaf tissue excised and mounted in sterile water, and gently covered with a coverslip. A flat area of pavement cells near the center of the leaf, at least 5 cells to the left or the right of the mid‐vein was chosen to image by brightfield to avoid imaging bias. Two separate fields of view (comprising different cells separated by at least 10 cells) focusing on the adaxial half of adaxial epidermal cells (or mesophyll cells in Figure 1C,D) were then imaged in Z‐series with between 6–10 focal planes imaged in 1 µm intervals. The complete mounting and imaging process for each leaf took less than 4 min. Plates were resealed and returned to growth chambers between samplings. Separate treatment conditions were imaged in series, such that one leaf from each experimental group was imaged before beginning again with the first treatment condition sampled. This was then repeated a minimum of 5 times for a total of 5 leaves from 5 separate plants imaged for each treatment condition. For mitochondria length measurements in roots, 1 image from each root zone defined in Figure 1J was obtained for each of 5 roots sampled from each treatment condition. Images were acquired alternatively from MZ to DZ then the reverse (DZ to MZ) for the next replication to minimize the effect of time under the coverslip on mitochondrial morphology.

For transiently transformed *Nicotiana* leaves, an 8 mm hole punch was used to isolate leaf discs from infiltrated areas, avoiding venation. One hole punch was collected from each transformed leaf and imaged 1 to 3 times.

For mitochondria movement analyses in Arabidopsis or transiently transformed tobacco leaves, the same imaging protocol was followed except the Z‐series acquired were 2 focal planes (1 µm interval) and were repeated 200 times in succession, with an individual frame time of 0.30 s (i.e. 1 Z‐series was acquired every 0.6 s for a total of 120 s recordings), 1–2 movies were obtained per leaf.

For Mitotracker Red (MTR; ThermoFisher Scientific, Waltham, MA, USA) staining and imaging, first true leaves were excised and cut in half to allow for dye penetration and immediately submerged in a solution of 300 µM MTR in sterile water with 0.01% Tween20 in 5 mL centrifuge tubes. Tubes containing leaves were wrapped in foil and left to stain tissues for 2 h without agitation. Initiation of staining was staggered such that each leaf imaged was incubated with stain solution for between 2 and 2.5 h to ensure replicability between experimental groups as extended incubation in MTR solution increases in mitochondria length (Figure S2C,D).

### 4.6 Spinning Disc Confocal Microscopy

Time lapse data for Figure 2E–G and Movies S1 and S2 was acquired on a Yokogawa W1 spinning disk microscope with an EM‐CCD camera (Hamamatsu 9100c; Hamamatsu Photonics, Hamamatsu City, Japan) on a Nikon Eclipse TE inverted stand equipped with a 100×/1.45 NA and standard GFP emission filters (Nikon, Tokyo, Japan). Arabidopsis plants expressing SHMT‐roGFP2 were processed and mounted using the same techniques as those for laser scanning confocal microscopy. Time lapse images were taken using the Micromanager v1.4 software, by taking sequential images of the adaxial surface of an epidermal cell with a frame time of 0.125 s and a total of 1000–3000 frames.

### 4.7 Transmission Electron Microscopy

Cotyledons from 11‐day‐old Arabidopsis seedlings were collected and immediately fixed in 2% paraformaldehyde, 2% glutaraldehyde and, 0.01% Tween 20 in 0.1 M sodium cacodylate buffer (pH 7.4). Samples were prepared for TEM following a previously described method [125], with modifications to improve the contrast based on sample incubation in heavy metals. After fixation, samples were kept in 2% osmium tetroxide (OsO_4_; Sigma–Aldrich, St. Louis, MO, USA) in water for 4 h at room temperature (RT) with agitation at 50 rpm. Samples were washed in water (3 × 30 min) followed by an incubation with 1% aqueous uranyl acetate (UAc) at 4°C overnight. The next day, samples were moved to 50°C for 2 h, washed three times in water, and incubated in a lead aspartate for 2 h at 50°C. The lead aspartate solution contained 0.04 g of L‐aspartic acid (Sigma–Aldrich, St. Louis, MO, USA) and 0.066 g of lead nitrate (Sigma–Aldrich, St. Louis, MO, USA) dissolved in 10 mL of water and pH adjusted to 5.5. Samples were washed at RT in water (3 × 30 min) and dehydrated in a series of anhydrous acetone (25%–50%–75%–95%–100%) at 4°C for 30 min each. Dehydrated samples were treated twice with 100% propylene oxide (EMS, Hatfield, PA, USA) for 30 min each. Samples were then embedded in Quetol resin (EMS, Hatfield, PA, USA). Resin infiltration was conducted at RT with agitation at 50 rpm over 7 days, according to the following ratios 3:1, 2:1, 1:1, 1:2,1:3 (v/v) propylene oxide: Quetol, each for ∼12–16 h. The last step in 100% Quetol with DMP‐30 (EMS, Hatfield, PA, USA) was repeated for 2 overnights at 4°C. Samples were then embedded in flat molds (EMS, Hatfield, PA, USA) with fresh resin and polymerized in a 60°C oven for 48 h.

Blocks were sectioned using a Leica Ultracut UCT (Leica Microsystems Inc., Buffalo Grove, IL, USA). 70 or 150 nm sections were collected and mounted on Formvar/Carbon‐coated gold slot grids. Tomograms were collected on 150‐nm thick sections previously incubated in 10 µl of 1/10 (v/v) 10‐nm colloidal gold particles as fiducials (AURION Immuno Gold Reagents & Accessories, Wageningen, Netherlands).TEM was performed using the Thermo Fisher Scientific Talos 120C G2 TEM in the Advanced Bioimaging Laboratory, operated at 120 kV and using a Ceta 16 M 4k × 4k CMOS camera with a pixel size of 0.833 nm. Dual‐axis tilt series were collected from −65° to +65° in 1° increments with Tomography software (version 5.13.0.5171REL, Thermo Fisher Scientific). The alignment of the tilt series and creation of stacks was conducted with Etomo in IMOD Tomography GUI (version 4.11.25) [126, 127]. The outer‐membrane, inner membrane, and cristae were annotated for 3D segmentation using DeepMIB [128], and imported into Dragonfly 2 (version 2022.2.0.1409, Comet Technologies, Montreal, Canada) for 3D visualization.

### 4.8 LC‐MS/MS Metabolite Measurements

For metabolite measurements of MEP RNAi lines, plants were treated with dexamethasone as described above. After which 150 mg of the shoot tissues were harvested and flash frozen in liquid nitrogen. For high light treated seedlings used for metabolite measurements, seedlings were treated with HL or kept in standard light for 3 h, then 150 mg of shoot tissues were harvested and flash frozen in liquid nitrogen. Frozen tissue was lyophilized and aliquoted in extraction tubes to 15 ± 2 mg dry weight per sample.

For extraction of hydrophilic metabolites, 3 steel beads (1 mm), 1 steel bead (3 mm), and 200 mg of 0.75‐1 mm glass beads were added before addition of 900 µL dichloromethane/ethanol (2:1, −80°C) followed by 150 µL dilute HCl (pH 1.7) and cryo‐extraction using a FastPrep24 system (3 × 20 s, Biomedicals Inc. Irvine, CA, USA). Phase separation was performed by centrifugation at 10 000 *g* for 2 min, and the upper phase was collected and placed on ice. Another 100 µL of dilute HCl was added to the extraction residue and the extraction and phase separation was repeated. Aqueous layers from each extraction were combined and stored at −80°C until analysis.

Separation of hydrophilic metabolites from the stored aqueous phase was performed by ion‐pairing chromatography on a Nucleoshell RP18 column (2.1 × 150 mm, particle size 2.1 µm, Macherey & Nagel, Düren, Germany) using a Waters ACQUITY UPLC System, equipped with an ACQUITY Binary Solvent Manager and ACQUITY Sample Manager (5 µL injection volume; Waters, Eschborn, Germany). Eluents A and B were aqueous 10 mmol/L tributyl amine (adjusted to pH 6.2 with glacial acetic acid) and acetonitrile, respectively. Elution was performed isocratically for 2 min with 2% eluent B, from 2 to 18 min with a linear gradient to 36% B, and from 18–21 min to 95% B, and isocratically from 21 to 22.5 min with 95% B, from 22.51 to 26 min eluent was returned to 2% B. The flow rate was set to 400 µL min^−1^, and the column temperature was maintained at 40°C.

Mass spectrometric analyses of small molecules were performed by targeted MS/MS via multiple reaction monitoring (MRM) by using a QTRAP 6500 (AB Sciex, Darmstadt, Germany) operating in negative ionization mode and controlled by Analyst 1.7.1 (AB Sciex, Darmstadt, Germany) (Table S4 [129]). The source operation parameters were the following: ion spray voltage, −4500 V; nebulizing gas, 60 psi; source temperature, 450°C; drying gas, 70 psi; curtain gas, 35 psi. Peak integration was performed using the MultiQuant software version 3.0.3 (Sciex, Toronto, CA).

### 4.9 Isolation of Epidermal and Mesophyll Cells

Epidermal and mesophyll isolation was performed essentially as described [130]. Rosette leaves from two‐week old Arabidopsis were placed between two tape strips. After gently peeling off the two tapes, the epidermal tissues on the abaxial side of the leaves were separated from the mesophyll cells and the adaxial epidermis. The adaxial tapes containing the mesophyll cells were incubated in protoplasting solution containing 20 mM MES (pH 5.7) 1% cellulase Onozuka R10, 0.25% Maceroenzyme R10 (both from Yakult Pharmaceutical Industry Co., Tokyo, Japan), 0.4 M mannitol, 10 mM CaCl_2_, 20 mM KCl, and 0.1% bovine serum albumin for 60 min at room temperature with agitation at 50 rpm. The resulting cell suspension containing mesophyll cells was collected and centrifuged at 100 *g* for 4 min at 4°C. The mesophyll cell pellet was washed twice with washing buffer (154 mM NaCl, 125 mM CaCl_2_, 5 mM KCl, 2 mM MES, pH 5.7) and collected. The abaxial tapes with the epidermal peels were washed twice with washing buffer and any vasculature was removed from the tape using forceps. The abaxial tapes containing epidermal cell samples and isolated mesophyll cells were frozen in liquid nitrogen and stored at −80°C prior to protein extraction.

### 4.10 Affinity Enrichment of Mitochondria

Epitope‐tagged mitochondria were rapidly isolated using co‐IP, as described previously [56]. All steps were carried out at 4°C. In brief, 1.5 g of leaves from 11‐day old Arabidopsis were harvested and ground gently in 3 mL KPBS (10 mM KH2PO4 pH 7.25, and 136 mM KCl) with mortar and pestle for 5 min. The resulting slurry was filtered through three layers of Miracloth using centrifugation at 2500 *g* for 5 min. The pellet containing cell debris and chloroplasts was discarded. This filtering process was repeated to produce a supernatant which included the mitochondria. Crude supernatant containing the mitochondria was incubated with 40 µL prewashed magnetic anti‐HA beads (ThermoFisher Scientific, Waltham, MA, USA) on an end‐over‐end rotator for 1 h in 1.5‐mL tubes. Magnetic beads were prewashed according to the manufacturer's instructions using KPBS. Magnetic beads were separated using a magnetic stand and washed five times with each 600 µL KPBS. Enriched mitochondria were eluted from beads by boiling for 10 min in 40 µL of Laemmli buffer (TBS pH 6.8, 5% 2‐mercaptoethanol, 2% SDS, 10% glycerol, 0.002% bromophenol blue) and used for immunoblotting and proteomics analysis.

### 4.11 Protein Extraction and Immunoblot Analyses

For isolated epidermal cells, tapes were held in liquid nitrogen in a mortar and pestle and adhered cells were scraped from the tape using a metal spatula then ground. For isolated mesophyll cells, total protein was extracted by grinding cell samples in the presence of liquid nitrogen. Then a membrane extraction buffer (MEB; 50 mM TRIS pH 7.5, 150 mM NaCl, 10% glycerol, 1% Nonidet P‐40, 0.5% deoxycholate, and 1x cOmplete Mini, EDTA‐free protease inhibitor cocktail) was added. Samples were centrifuged at 16 000 *g* and supernatants (total protein extracts) were transferred to new tubes and protein concentrations determined by Bio‐Rad Protein Assay according to the manufacturer's instructions (Bio‐Rad, Hercules, CA, USA). Equal amounts of total protein were added to Laemmli buffer and boiled for 10 min.

Total protein samples and a portion of the eluted samples of affinity enriched mitochondria boiled in Laemmeli buffer were centrifuged at 16000 *g* and supernatants transferred to new tubes. Proteins were separated on a 10% w/v SDS‐PAGE gel, then transferred to polyvinylidene difluoride membranes (Millipore, Burlington, MA, USA). Membranes were probed with mouse anti‐GFP (Roche #11814460001, Basel, Switzerland), rabbit anti‐isocitrate dehydrogenase (IDH, Agrisera #AS06‐203A, Vännäs, Sweden), rabbit anti‐cytochrome oxidase subunit 2 (COX2, Agrisera #AS04‐053A, Vännäs, Sweden), rabbit anti‐catalase (CAT, Agrisera #AS09‐501, Vännäs, Sweden), and rabbit anti‐luminal binding protein (BIP Agrisera #AS09‐481, Vännäs, Sweden) antibodies at dilutions of 1:3000, 1:5000, 1:1000, 1:1000. And 1:2000 respectively. Anti‐mouse (Bio‐Rad #L00560A, Hercules, CA, USA) and anti‐rabbit (Sigma–Aldrich #A0545, St. Louis, MO, USA) secondary antibodies conjugated to horseradish peroxidase were then used at 1:5000 dilutions. Chemiluminescent detection of secondary antibodies was performed with the SuperSignal West Duration substrate (ThermoFisher Scientific, Waltham, MA, USA) according to the manufacturer's instructions.

### 4.12 LC‐MS/MS Proteomics

For proteomics analysis, the eluted enriched mitochondria protein samples were transferred to a separate centrifuge tube. At room temperature, 2–3 mL of 8 M urea was added to each tube, and centrifuged at 4400 rpm for 30 min (repeated 3 times). The protein was reduced by DTT (20 mM working concentration) for 60 min at 37°C and alkylated by IAA (50 mM working concentration) avoiding light for 0.5 h at room temperature. Samples were then buffer exchanged to 50 mM ammonium bicarbonate using an Amicon Ultra‐4 10 K. The solution was then incubated with trypsin (trypsin: protein = 1: 50, wt/wt) at 37°C overnight. The solution was desalted using a C18 Zip‐Tip (Waters). Finally, the tryptic digest was lyophilized by SpeedVac and stored at −80°C.

The tryptic digests were resuspended in 20 µL of 0.1% formic acid in water. Separated by Nano‐LC and analyzed by on‐line electrospray tandem mass spectrometry. The experiments were performed on a Vanquish Neo chromatography system (ThermoFisher Scientific, Waltham, MA, USA) connected to a quadrupole Orbitrap Eclipse Tribrid Mass Spectrometer equipped with an EASY‐Spray ion source. 5 µL of peptide sample was loaded onto the trap column (Acclaim PepMap C18, 75 µm × 2 cm; ThermoFisher Scientific, Waltham, MA, USA) with a flow of 10 µL min^−1^ for 3 min and subsequently separated on the analytical column (Acclaim PepMap C18, 75 µm × 25 cm, ThermoFisher Scientific, Waltham, MA, USA) with a linear gradient, from 4% B to 25% B in 100 min, then from 25% B to 45% B in 15 min. The column was then re‐equilibrated at initial conditions for 5 min. The flow rate was maintained at 300 nL min^−1^ and column temperature was maintained at 45°C. The electrospray voltage of 1.7 kV versus the inlet of the mass spectrometer was used. Nano‐LC separation parameters: A‐ water, 0.1% formic acid; B‐ 80% acetonitrile 0.1% formic acid over a C18 column with a flow rate of 300 nL min^−1^. Separation was performed over 120 min, starting at 4% B, at 100 min 25% B, at 115 min 45% B, at 115.1 min 97% B, 120 min 97% B.

The Orbitrap Fusion Mass Spectrometer was operated in data‐dependent mode to switch automatically between MS and MS/MS acquisition. Survey full‐scan MS spectra (m/z 375–1500) was acquired with a mass resolution of 240 K, followed by fifteen sequential high energy collisional dissociations (HCD). The AGC target was set to 10.0 × 10^5^, and the maximum injection time was 100 milliseconds. MS/MS acquisition was performed in ion trap mode. The AGC target was set to 3 × 10^4^, and the isolation window was 1.6 m z^−1^. Ions with charge states 2+ to 6+ were sequentially fragmented by higher energy collisional dissociation (HCD) with a normalized collision energy (NCE) of 35%, fixed first mass was set at 100. In all cases, one micro scan was recorded using dynamic exclusion of 30 s.

The raw data was processed using MaxQuant (version 2.1.4.0) with a homemade database for protein identification and quantification utilizing the Label‐Free Quantification (LFQ) algorithm. Mass tolerances for precursor and fragment ions were 6 and 10 ppm, respectively, the minimum peptide length was 6 amino acids, and the maximum number of missed cleavages for trypsin was 2.

### 4.13 Quantification and Statistical Analysis

Quantification of mitochondria lengths were performed in ImageJ‐Fiji. Each Z‐series was processed into a max intensity projection, and a minimum of 40 individual mitochondria were sampled in each image in a diagonal line beginning at the top left of the image to the bottom right. Each mitochondria sampled was measured by manually tracing a segmented line over the length of the mitochondria. Quantification of mitochondria length in tobacco transient assays were performed using the mito‐mCherry signal to maintain parity between different constructs. Quantification of cross‐sectional mitochondria area and circularity was manually performed using the ImageJ wand tool, using individual focal planes instead of max intensity projection to ensure that mitochondria sampled were in the same focal plane as mesophyll cell chloroplasts and not at the abaxial side of the adaxial epidermal cells. Quantification of mitochondrial motility was performed using the TrackMate ImageJ plugin on threshold segmented time lapse images. TrackMate data for each timelapse was exported and average mitochondria velocity for all mitochondria in the timelapse calculated in Excell. Quantification of relative NADH/NAD+ levels in plants expressing the Peredox‐mCherry were performed by subtracting the sum signal intensity of the 400–500 nm collected emission spectra (background) from the sum signal intensity of the 400–530 nm emission spectra to determine the tSapphire fluorescence intensity (500–530 nm), then dividing the resulting value by the sum signal intensity of mCherry for an individual image.

ER morphology assessment was performed on regions of static cortical ER (i.e. areas without cytoplasmic streaming events), by isolating 1–3 ROIs from different cells in each image (See Figure S8), then each individual ROI was processed using the AnalyzER program to determine average tubule length, cisternal area, and polygonal area of each ROI.

All experiments involving measurements and quantifications from images were repeated at least two times with similar results. Statistical analysis of quantified data was performed using previously published methods [131]. Relevant one‐way or two‐way between subjects type II ANOVA were conducted as needed to test the effects of experimental variables, type III ANOVA were used for unbalanced data sets. For each dataset the Shapiro‐Wilk test, Breusch‐Pagan test, and Levene's test were used to test the ANOVA normality (Shapiro‐Wilk, Breusch‐Pagan) and homogeneity of variance assumptions (Levene's). In most cases the homogeneity of variance assumption was violated due to the increased variance in mitochondrial length after high light treatment. If the assumption tests were violated (*p* < 0.05), Box‐Cox or log transformations were applied to the dataset and the ANOVA was rerun using transformed data. If assumption tests were still violated, then weighted least squares regression was applied to the ANOVA model (referred to in the text as a wANOVA). All datasets which were subjected to weighted least squares regression passed the residual analysis. From the appropriate model for each respective data set post‐hoc pairwise comparisons (CRAN R package *emmeans*) were then used with Tukey adjustment applied. Summarized statistical results for each experiment can be found in the graphical representations of the data, with a brief description of statistical tests performed in the relevant figure legends. Full data descriptions for each experiment processed using these methods, including *n* values, descriptions of what *n* values represent, and the full results of the statistical tests can be found in Table S1, the α‐value for each test described in Table S1 was 0.05.

For analysis of proteomics data, MaxQuant results were imported into Perseus (v2.0.11) for data processing, statistical analysis, and visualization. The initial dataset was log_2_‐transformed, filtered to remove protein groups of potential contaminants, reverse hits, and proteins only identified by site. Further filtering was performed such that only proteins containing valid LFQ values greater than 66% in all replicates were retained. Then missing values were imputed using random numbers generated from an ideal gaussian distribution (width = 0.3, down shift = 1.8), and the data set normalized by Z‐score (by columns). Finally, the differential abundance of log_2_ protein LFQ intensities was calculated and volcano plots generated for each comparison. Comparisons were performed between the HL and control groups, as well as between fumarate‐treated and control conditions. Statistical significance was determined using Student's t‐tests, with an S_0_ value of 0.5 applied to stabilize the test statistic and improve the reliability of detecting significant changes. A false discovery rate (FDR) threshold of 0.05 was used to account for multiple testing. The two curved lines (See Figure 6C) represent threshold boundaries for statistical significance and fold change, visually identifying differentially expressed proteins.

For analysis of metabolite quantification data, the average integrated peak area values of the two groups were log‐transformed, and the fold change was calculated by subtracting treatment conditions (DEX or HL) from control conditions. Statistical significance was determined using Student's t‐tests, with a false discovery rate (FDR) threshold of 0.05 used to account for multiple testing.

## Author Contributions

K.D., H.C., and E.A designed the research and prepared the manuscript. E.A. performed most of the experiments and analyzed data, with assistance from J.G., E.S, and M.P., W.V. produced the MEP pathway mutants. A.Z. and T.B.S. performed the TEM and electron tomography imaging. H.C. designed and performed proteomics with Q.Z., and W.V, G.B., A.T., and M.H performed metabolite measurement and analysis.

## Ethics Statement

This study does not involve human participants or animals and therefore did not require ethics approval.

## Conflicts of Interest

The authors declare no conflicts of interest.

## Supporting information




**Supporting File 1**: advs74002‐sup‐0001‐SuppMat.docx.

**Supporting File 2**: advs74002‐sup‐0002‐TableS1‐S4.zip.

**Supporting File 3**: advs74002‐sup‐0003‐MovieS1‐S6.zip.

## Data Availability

Summary of measurements and statistical test information are provided in Table S1. This study generated a proteomics data set provided in Table S2. No unique code was generated. All datasets generated by TEM were uploaded to the EMPIAR databank—(Accession number EMPIAR‐47486939).
