# Supplementary material for: Chloroplast Stress Signals Orchestrate Epidermis‐Specific Remodeling of Mitochondria and ER Under High Light
Source: Adv Sci (Weinh). 2026 Jan 27;13(18):e14970. doi: 10.1002/advs.202514970 (PMC13042555; doi:10.1002/advs.202514970)
Supplement: Supplementary file 1 — Supporting File 1: advs74002‐sup‐0001‐SuppMat.docx. [file ADVS-13-e14970-s001.docx]

**Chloroplast Stress Signals Orchestrate Epidermis-Specific Remodeling of Mitochondria and ER under High Light**

Evan R. Angelos^1‡^, Hee-Seung Choi^1‡†^, Jingzhe Guo^1^, Andrea A. Zanini^2^, Tessa M. Burch-Smith ^2^, Emily Snyder^1,3^, Matthew Part^1,4^, Wilhelmina van de Ven^1^, Manhoi Hur^1^, Gerd Ulrich Balcke^5^, Alain Tissier^5^, Quanqing Zhang^1,6^, Katayoon Dehesh^1*^

^1^ Institute for Integrative Genome Biology and Department of Botany and Plant Sciences, University of California, Riverside, CA 92521

^2^ Donald Danforth Plant Science Center, St. Louis, MO 63132

^3^Department of Biology, West Chester University of Pennsylvania, PA, 19383.

^4^ Department of Plant Biology, University of California, Davis, CA 95616

^5^ Leibniz Institute of Plant Biochemistry, Department of Cell and Metabolic Biology; Weinberg 3, D-06120 Halle (Saale), Germany

^6^ Proteomics Core Facility, Institute for Integrative Genome Biology and Department of Botany and Plant Sciences, University of California, Riverside, CA 92521

† Current affiliation: Department of Life Sciences, Pohang University of Science and Technology, Pohang, Republic of Korea

^‡^ Authors contributed equally to this work.

*Corresponding Author:

Katayoon Dehesh

Email: katayoon.dehesh@ucr.edu

**Supplemental Figure Legends**

**
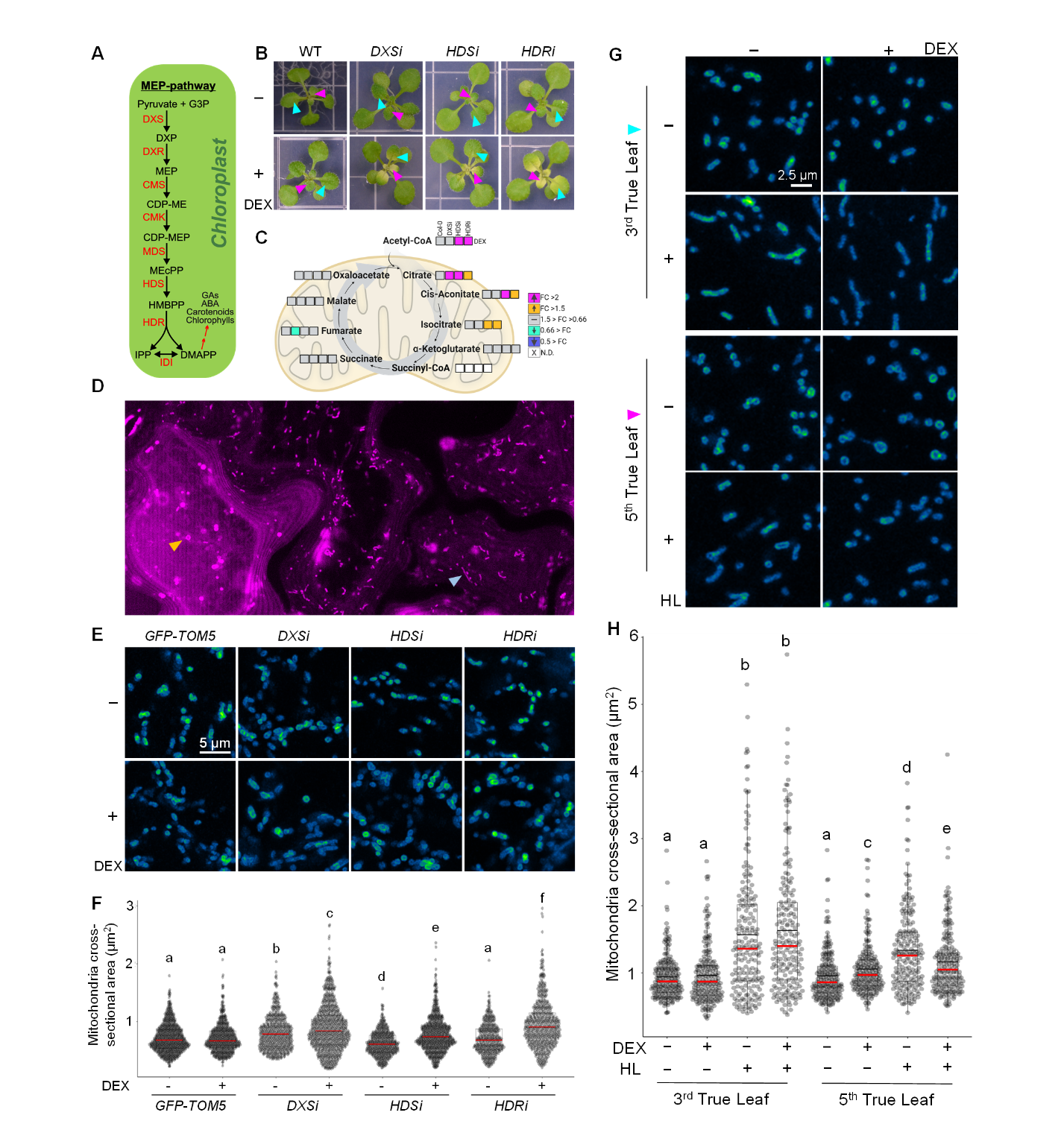
**

**Figure S1. MEP Pathway Perturbation Alters TCA Cycle Metabolite Levels and Mitochondrial Morphology**

**(A)** Schematic representation of the MEP pathway, which is responsible for the biosynthesis of isoprenoids in plastids. Pathway intermediates are labeled in green: DXP (1-Deoxy-D-xylulose 5-phosphate), MEP (2-C-methyl-D-erythritol 4-phosphate), CDP-ME (4-(cytidine 5'-diphospho)-2-C-methyl-D-erythritol), CDP-MEP (2-Phospho-4-(cytidine 5'-diphospho)-2-C-methyl-D-erythritol), MEcPP (2-C-methyl-D-erythritol-2,4-cyclodiphosphate), HMBPP ((E)-4-hydroxy-3-methylbut-2-enyl diphosphate), IPP (isopentenyl diphosphate), and DMAPP (dimethylallyl diphosphate). Enzymes catalyzing each step are labeled in red: DXS (1-deoxy-D-xylulose-5-phosphate synthase), DXR (1-deoxy-D-xylulose-5-phosphate reductoisomerase), CMS (4-(cytidine 5'-diphospho)-2-C-methyl-D-erythritol synthase), CMK (4-(cytidine 5'-diphospho)-2-C-methyl-D-erythritol kinase), MDS (2-C-methyl-D-erythritol 2,4-cyclodiphosphate synthase), HDS (1-hydroxy-2-methyl-2-butenyl 4-diphosphate synthase), HDR (1-hydroxy-2-methyl-2-butenyl 4-diphosphate reductase), and IDI (isopentenyl-diphosphate isomerase).

**(B)** Representative images of transgenic plants harboring a DEX-inducible RNAi construct targeting *DXS*, *HDS*, and *HDR*, captured before (-) and 72 hours after DEX (+) treatment. Chlorotic leaf emergence is observed following induction. Magenta and cyan arrows mark representative regions on the 3^rd^ and 5^th^ true leaves for confocal imaging.

**(C)** Schematic representation of the TCA cycle, depicting the levels of intermediates in wild-type (WT) and RNAi transgenic lines (*DXSi*, *HDSi*, and *HDRi*), highlighting the differential accumulation of selected intermediates between RNAi lines and WT.

**(D)** Confocal imaging of mitochondria stained with MitoTracker Red, revealing staining-induced artifacts leading to apparent mitochondrial enlargement. Yellow arrow highlights swollen mitochondria, white arrow highlights elongated mitochondria.

**(E)** Transgenic RNAi lines introgressed into *3x-HA-sGFP-TOM5* (hereafter *GFP-TOM5*) line enable a more accurate assessment of mitochondrial morphology. Mitochondrial size was measured before and 72-h after DEX treatment, showing a significant increase across all RNAi lines. Scale bars = 5 µm.

**(F)** Quantification of mitochondrial cross-sectional area in fifth true leaves under MEP pathway perturbation reveals significant differences among treatments. Letters denote statistically distinct groups: two-way wANOVA; Tukey’s post-hoc test; n = 401-751 mitochondria; α = 0.05.

**(G–H**) Representative confocal images and quantification of mitochondrial length in HDSi plants treated with DEX or mock solution and maintained under standard light conditions for 66 hours. Following this period, seedlings were either exposed to HL for 6 hours or kept under standard light as controls. Scale bars = 2.5 µm. Letters denote statistically distinct groups: two-way wANOVA; Tukey’s post-hoc test; n = 200 mitochondria; α = 0.05.

**
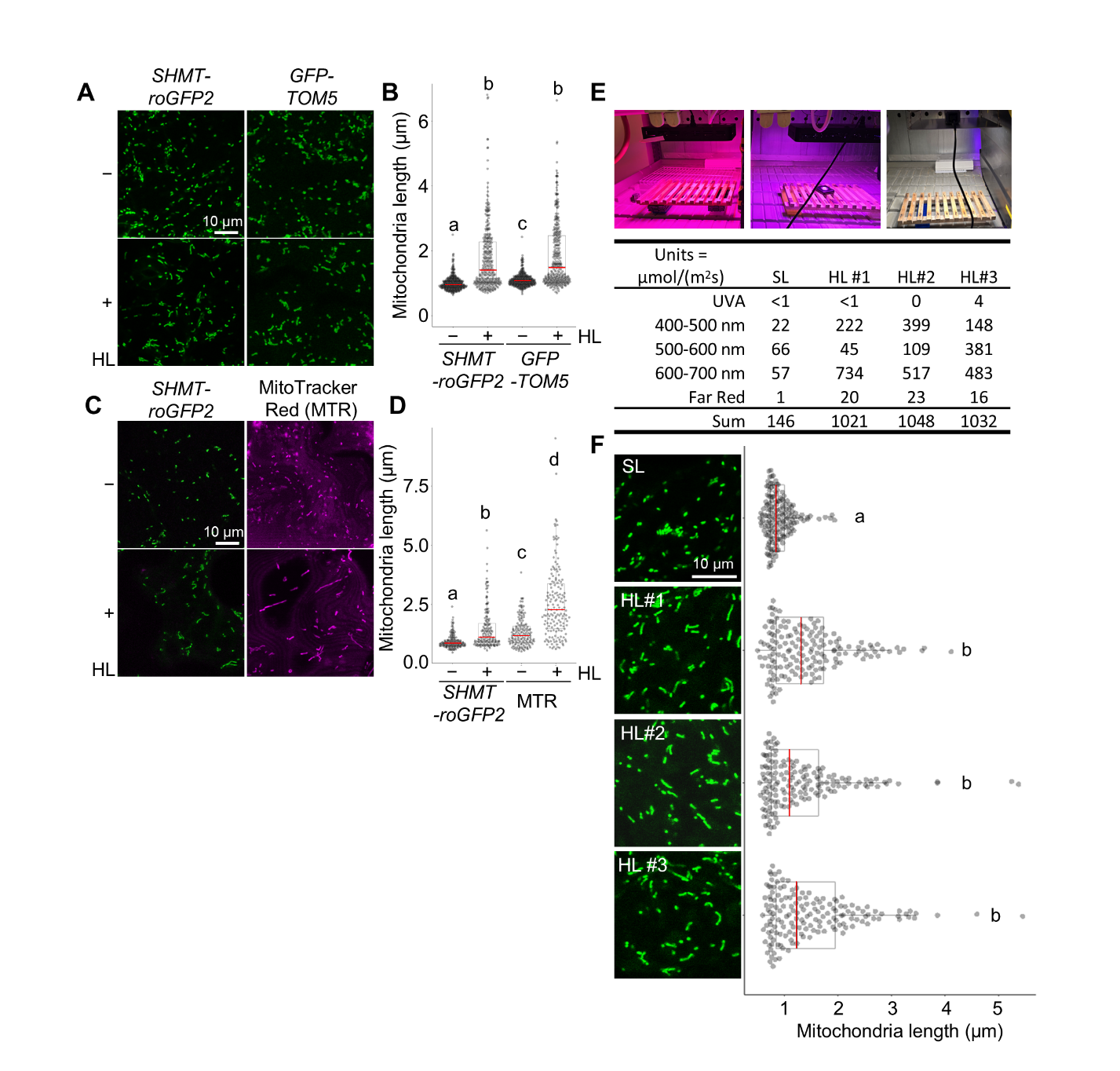
**

**Figure S2. High Light Treatment Increases Mitochondrial Size in Epidermal Cells, Independent of Mitochondrial Marker or Wavelength of Light Exposure**

**(A)** Representative confocal images of mitochondria in epidermal cells expressing either GFP-TOM5 or SHMT (Serine Hydroxymethyltransferase)-roGFP2 under standard light (HL-) and high light (HL+) conditions. Scale bars = 10 µm.

**(B)** Quantification of mitochondrial length in epidermal cells under standard light (HL-) and high light (HL+) conditions, showing a significant increase in mitochondrial size upon HL exposure in both SHMT-roGFP2 and GFP-TOM5 transgenic lines. Letters denote statistically distinct groups: two-way wANOVA; Tukey’s post-hoc test; n = 320 mitochondria; α = 0.05.

**(C-D)** Confocal images (**C**) and quantification (**D**) of mitochondrial length in *SHMT-roGFP2* transgenic lines and MitoTracker Red (MTR)-stained plants under standard light (HL-) and high light (HL+) conditions, MTR staining served as an additional control to validate that high light-induced mitochondrial elongation is not caused by an interaction between high light treatment and overexpression of genetically encoded mitochondrial markers. Letters denote statistically distinct groups: two-way wANOVA; Tukey’s post-hoc test; n = 160 mitochondria; α = 0.05.

**(E)** Spectral distribution of different high light treatments, displaying the intensity of UVA, blue, green, red, and far-red light used in the experiment.

**(F)** Confocal images and quantification of mitochondrial length in plants exposed to SL and different HL spectrums (HL #1, HL #2, HL #3), demonstrating that mitochondrial elongation occurs regardless of the specific wavelength applied. Quantification confirms the consistent increase in mitochondrial size in response to HL treatment. Letters denote statistically distinct groups: one-way wANOVA; Tukey’s post-hoc test; n = 160 mitochondria; α = 0.05.

**
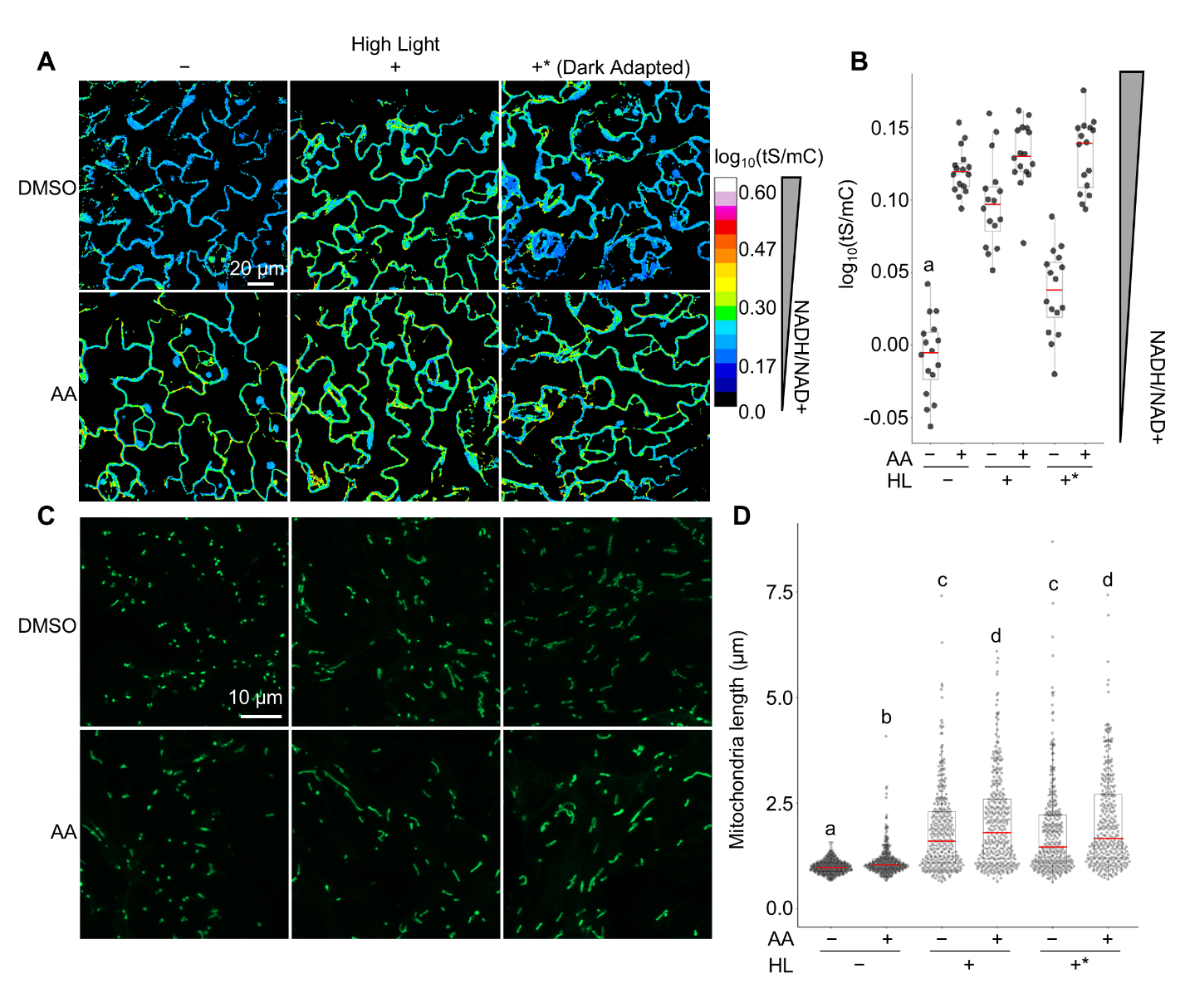
**

**Figure S3. Light Intensity and Antimycin A treatment redundantly increase cytosolic NADH/NAD+ ratio.**

**(A)** Ratiometric confocal images of epidermal cells from plants expressing the cytosolic, genetically encoded NADH/NAD+ sensor Peredox-mCherry. The log_10_ ratio of tSapphire (NADH binding increases tSapphire fluorescence intensity) to mCherry fluorescence is shown (log_10_ tS/mC). Seedlings were treated with DMSO (control) or the mitochondrial electron transport inhibitor Antimycin A (AA), followed by exposure to high light (HL+) for 12 hours, standard light (HL−) for 12 hours, or high light for 12 hours followed by 3 hours of darkness (+*; dark adapted). Scale bar: 10 µm.

**(B)** Quantification of log_10_ tS/mC from fluorescence images in (A) demonstrates the redundant effects of light and AA treatment on cytosolic NADH levels, validating the effectiveness of AA spray treatment on epidermal cells. Letters denote statistically distinct groups: two-way ANOVA; Tukey’s post-hoc test; n = 16 images; α = 0.05.

**(C-D)** Representative confocal images of mitochondria in epidermal cells of plants expressing SHMT-roGFP2 included in the same experiment as (A). Scale bars = 10 µm. Quantification of mitochondrial length demonstrates that HL and AA treatments, including the dark adaptation treatment after HL treatment maintained the observed mitochondria length phenotypes as in Fig. 4 (E-H) during the validation of AA spray treatment. Letters denote statistically distinct groups: two-way wANOVA; Tukey’s post-hoc test; n = 320 mitochondria; α = 0.05.

**
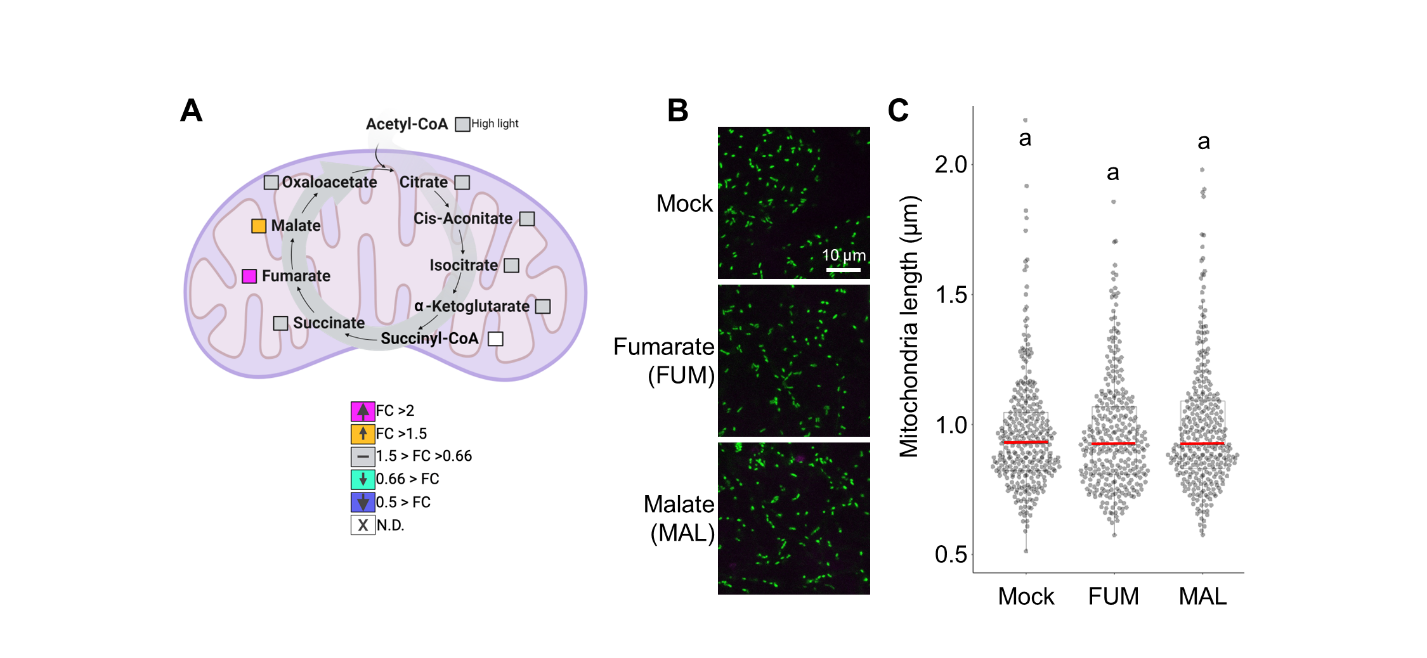
**

**Figure S4. Accumulation of TCA Metabolites does not Induce Mitochondrial Elongation**

**(A)** Metabolic profiling of TCA cycle intermediates in high light (HL) treated plants compared to non-treated plants, showing increased abundance of fumarate (FUM) and malate (MAL).

**(B)** Representative confocal images of mitochondria in epidermal cells of plants treated with exogenous FUM and MAL, compared to the mock control. Scale bars = 10 µm.

**(C)** Quantification of mitochondrial length in epidermal cells after FUM and MAL treatment shows no significant difference from the mock control, indicating that TCA metabolite accumulation alone does not drive mitochondrial elongation. Letters denote statistically distinct groups: one-way wANOVA; Tukey’s post-hoc test; n = 320 mitochondria; α = 0.05.

**
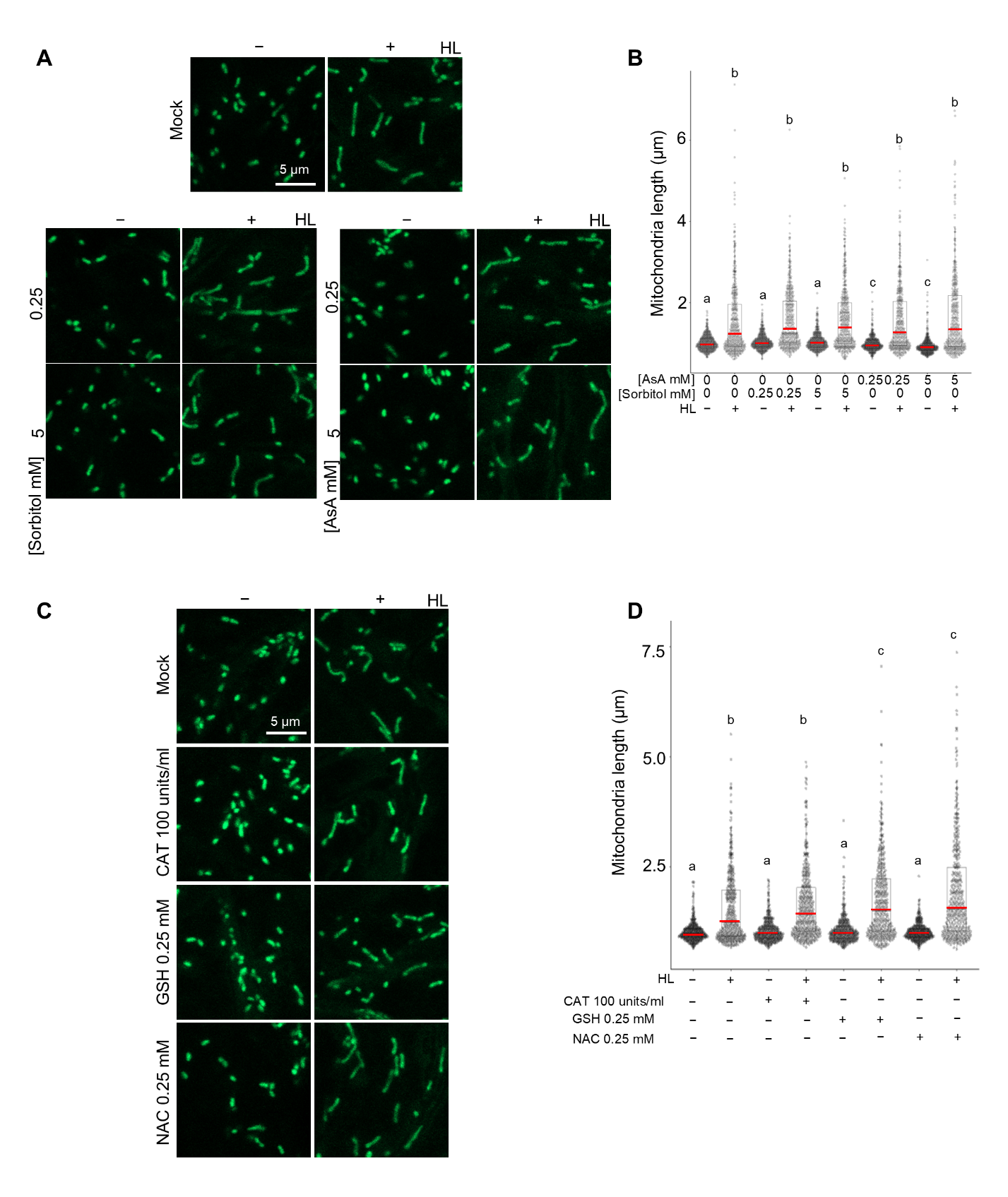
**

**Figure S5. Exogenous antioxidant pretreatment does not affect HL-induced mitochondrial elongation.**

**(A–B)** Representative confocal images and quantification of mitochondrial length in epidermal cells of plants pretreated with ascorbic acid (AsA) or sorbitol (negative control) at the indicated concentrations for 2 h prior to high light (HL) exposure for 6 h. Letters denote statistically distinct groups: two-way wANOVA; Tukey’s post-hoc test; n = 400 mitochondria; α = 0.05.

(**C–D**) Representative confocal images and quantification of mitochondrial length in plants pretreated for 2 h with catalase (CAT, 100 units mL⁻¹), reduced glutathione (GSH, 0.25 mM), or N-acetylcysteine (NAC, 0.25 mM) before HL exposure for 6 h. Scale bars = 5 µm. Letters denote statistically distinct groups: two-way wANOVA; Tukey’s post-hoc test; n = 400 mitochondria; α = 0.05.

**
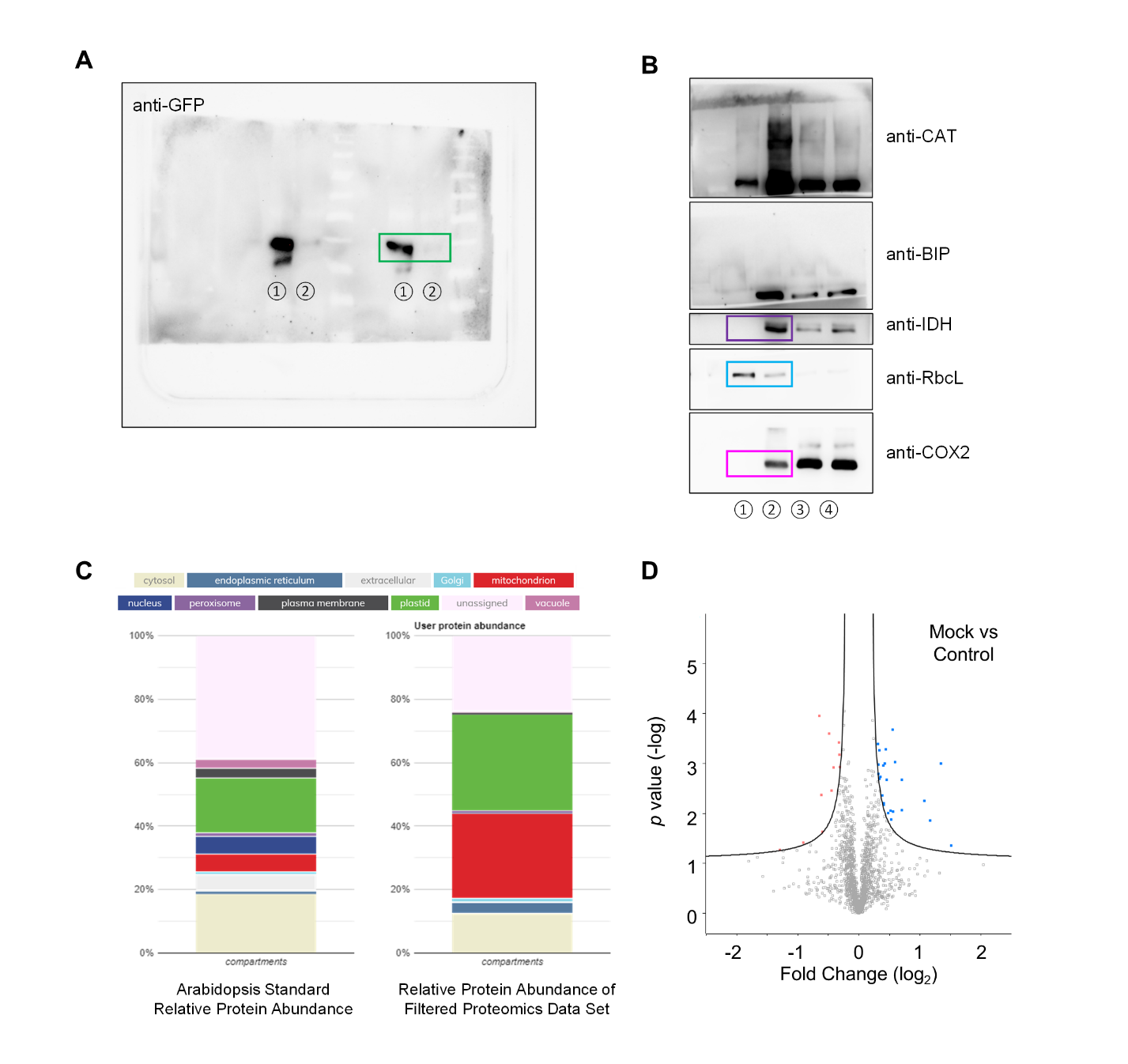
**

**Figure S6. Relative Enrichment of Mitochondria Samples from *pATML1:GFP-TOM* lines**

**(A)** Blot of GFP-TOM5 expression in epidermal and mesophyll cells using GFP antibodies. Green outline highlights area shown in Fig. 6B. Lane 1: epidermal cell total protein, Lane 2: mesophyll cell total protein. **(B)** Blots of partial gels showing CAT (catalase; peroxisomal marker), and BIP (ER marker), IDH, RbcL, and COX2 protein levels, in total lysates and enriched mitochondria samples after GFP purification. Purple outline highlights the IDH lanes, magenta outline highlights the COX2 lanes, and blue outline highlights the RBCL lanes shown in Fig. 6B. Lanes shown: 1 total lysate of control sample 1; 2 mitochondria enriched control sample 1; 3 mitochondria enriched control sample 2; 4 mitochondria enriched high light sample 1. shown Fig. 6B. Red box (1) outlines a duplicate blot of the COX2 blot (magenta).

**(C)** SUBA5 was used to determine the relative localization enrichment of the list of all protein groups detected in our filtered proteomics data set (1771 proteins; Table S2), compared to the relative abundance of the standard set of known Arabidopsis proteins, suggesting a relative enrichment in mitochondrial proteins.

**(D)** Volcano plot depicting differential protein abundance in mitochondrial-enriched samples between additional mock treatment conditions and control plants. The curved lines represent threshold boundaries for statistical significance and fold change, visually identifying differentially expressed proteins (n=3, using Student’s t-tests, with an S₀ value of 0.5 applied to stabilize the test statistic, FDR threshold =0.05).

**
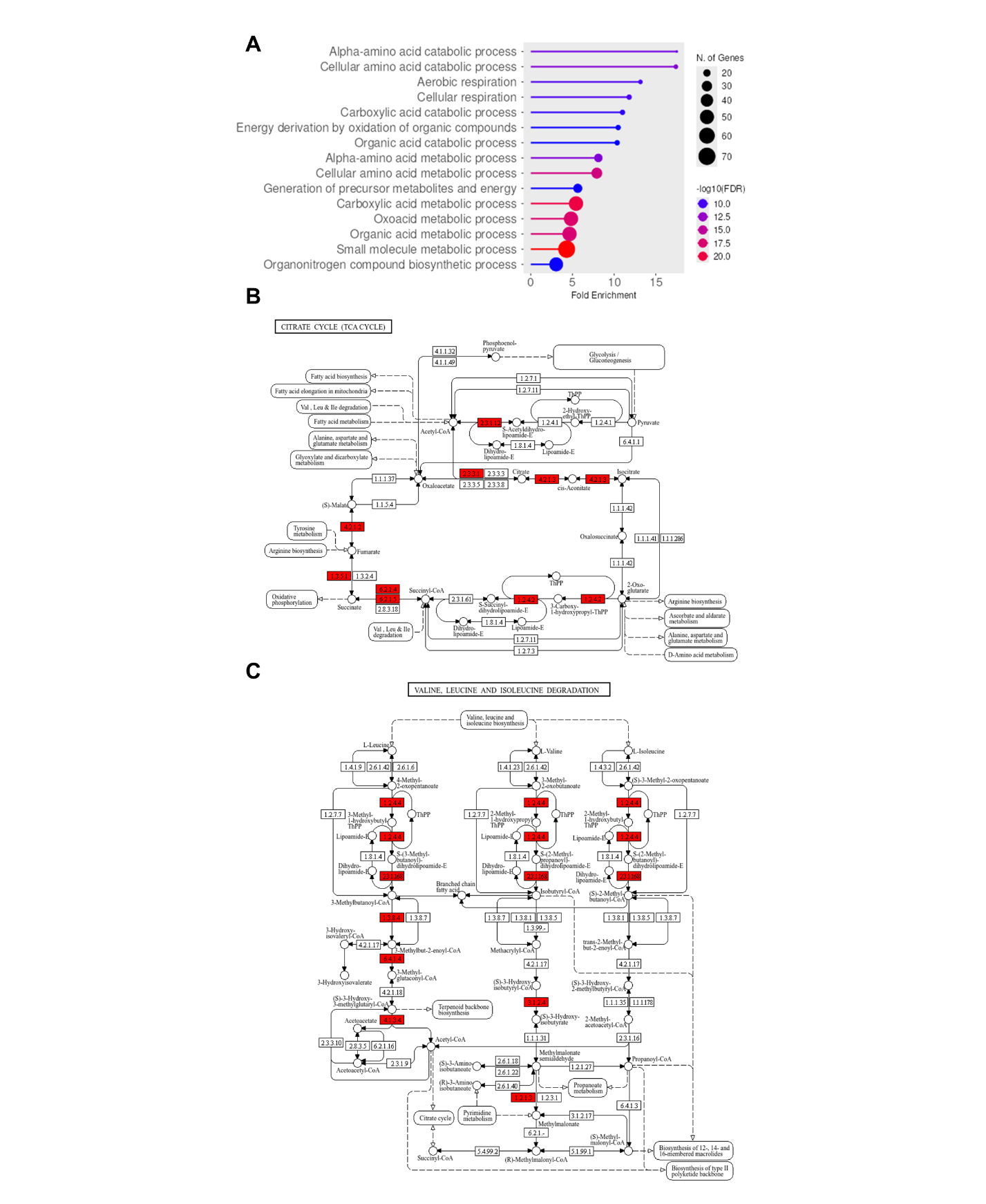
**

**Figure S7. High Light Treatment Increases the Abundance of Enzymes in the Citric Acid Cycle and Amino Acid Degradation Machinery**

**(A)** Bubble plot representation of biological process GO term enrichment analysis performed on 248 proteins with increased abundance specifically in HL treatment conditions, highlighting the most significantly affected pathways under high light (HL) conditions. The bubble size represents the number of proteins involved in each pathway, while the color gradient indicates statistical significance (-log_10_ [p-value]).

**(B-C)** KEGG pathway representation depicting differentially abundant mitochondrial enzyme activities which were enriched in mitochondria after HL treatment. (B) TCA cycle KEGG pathway representation, showing an increased abundance of enzymes involved in mitochondrial energy metabolism. (C) Pathway diagram illustrates the upregulation of enzyme activities associated with branched chain amino acid degradation pathways in mitochondria under HL exposure.

**
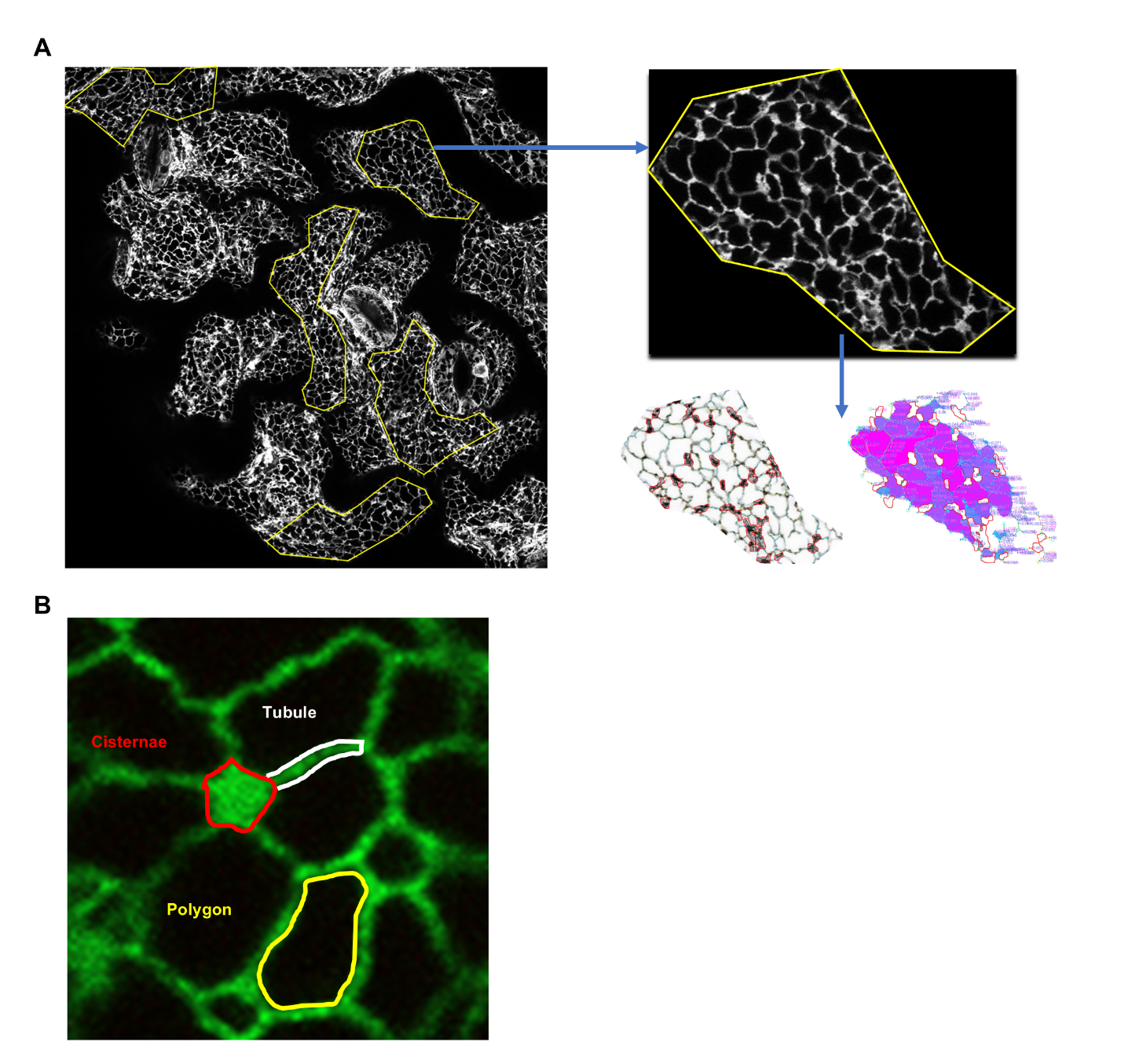
**

**Figure S8. Substructural Organization of ER Morphology**

**(A)** Depiction of the methodology used to analyze ER morphology using the AnalyzER program. A z-series was used to create max intensity projection wherein contiguous areas of the cortical ER network were divided into ROIs (examples outlined in yellow) that did not contain any cytoplasmic streaming events, which can be misidentified by the AnalyzER program as cisternae. These ROIs were isolated, and ER morphological traits identified and quantified by the AnalyzER program.

**(B)** Visualization of ER substructures highlighting cisternae (red box), tubules (outlined in white), and polygons (empty space between tubules and cisternae outlined by yellow boundaries), illustrating their organization and spatial arrangement.

**
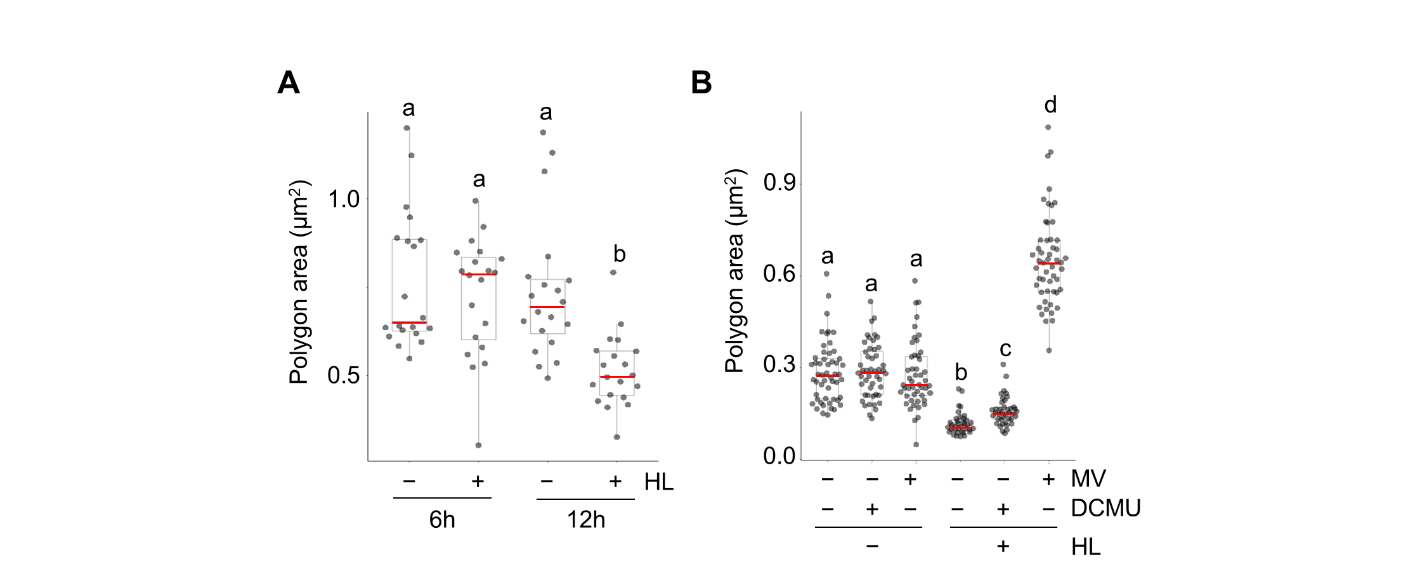
**

**Figure S9. Functional Chloroplasts Drive High Light-Triggered Reduction in ER Polygon Size**

**(A)** Quantification of mean ER polygon area in plants exposed to standard light (HL-) or 6 and 12 hours of high light (HL+) treatment, expanded data from the experiment shown in Fig. 7 (A-B). Letters denote statistically distinct groups: two-way ANOVA; Tukey’s post-hoc test; n = 20 cells; α = 0.05.

**(B)** Quantification of mean ER polygon area in plants exposed to high light (HL+) or standard light (HL-) with or without DCMU or MV pretreatment, expanded data from the experiment shown in Fig. 7 (C-D). Letters denote statistically distinct groups: two-way type III ANOVA; Tukey’s post-hoc test; n = 36-52 cells; α = 0.05.

**
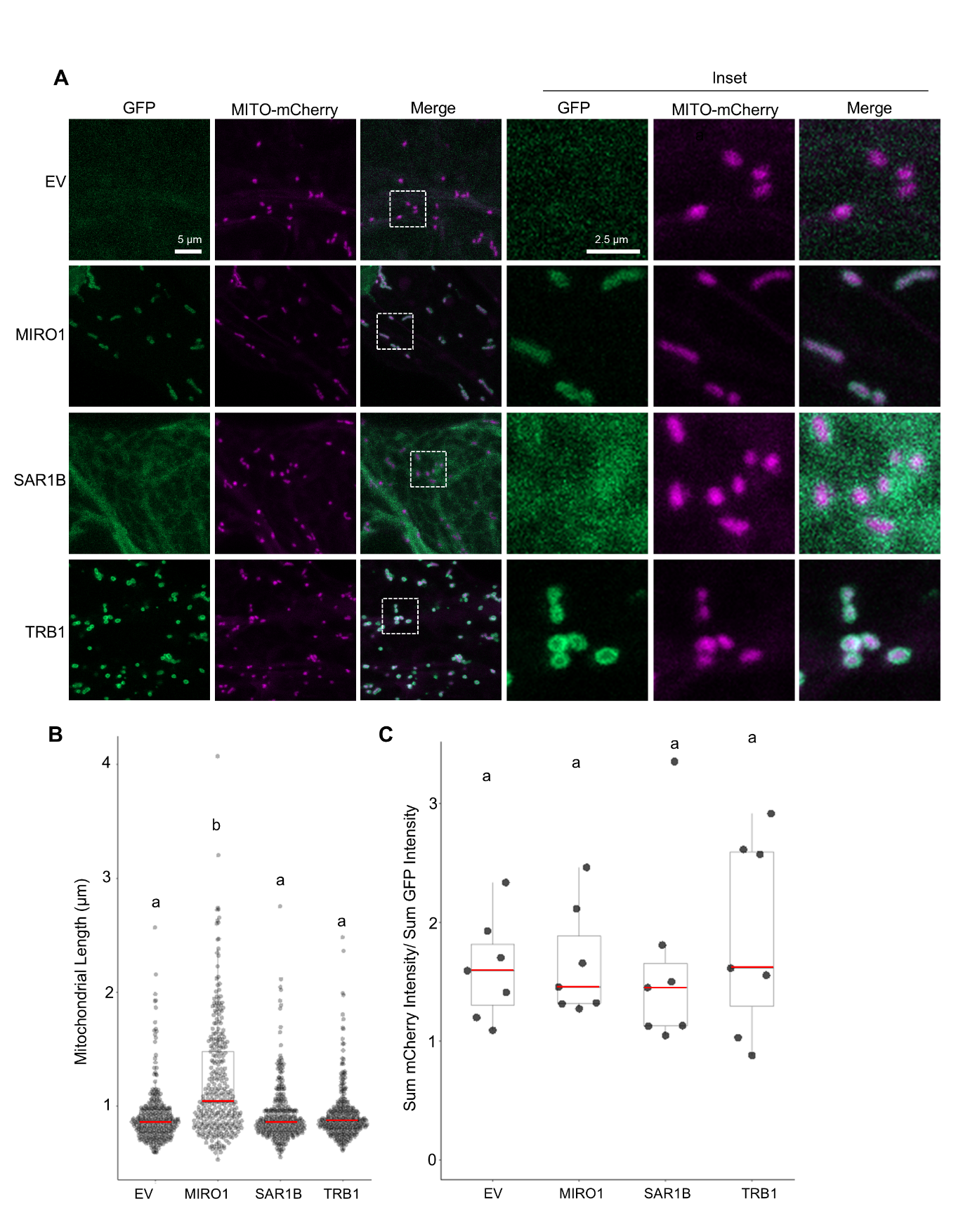
**

**Figure S10. MIRO1 Overexpression Alters Mitochondrial Morphology**

**(A)** Representative confocal images of mitochondrial morphology in transiently transformed tobacco leaves overexpressing an empty vector (EV, control) or N-terminally, GFP-tagged MIRO1, SAR1B, and TRB1. MITO-mCherry was used to confirm mitochondrial identity, as shown in the merged GFP and MITO-mCherry images. The magnified view (inset) within the dotted white square highlights alterations in mitochondrial morphodynamics in response to protein overexpression. Primary image scale bar = 5 µm, inset scale bar = 2.5 µm.

**(B)** Quantification of mitochondrial length from the images shown in (A), demonstrating increased mitochondrial length only in response to overexpression of MIRO1. Letters denote statistically distinct groups: one-way wANOVA; Tukey’s post-hoc test; n = 320 mitochondria; α = 0.05.

**(C)** The relative ratio of mCherry intensity to GFP intensity confirms comparable protein expression levels across constructs in the transient assay. Letters denote statistically distinct groups: one-way wANOVA; Tukey’s post-hoc test; n = 8 images; α = 0.05.

**
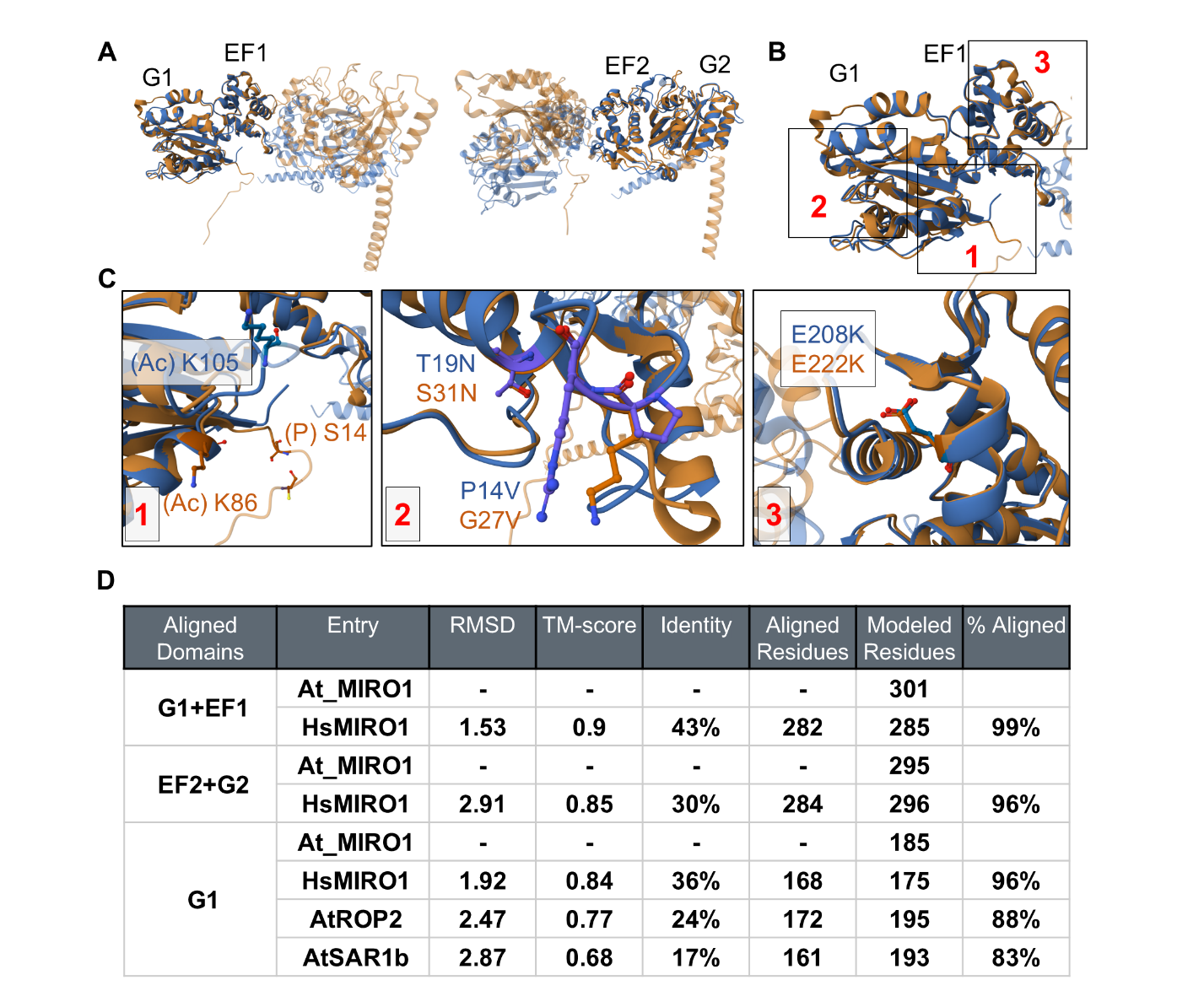
**

**Figure S11. Structural Alignment of AlphaFold3 MIRO1 Models and Conservation of Key Residues, related to Figure 9**

**(A-B)** Structural alignment of AlphaFold-predicted Arabidopsis MIRO1 (orange) and human MIRO1 (blue), highlighting strong structural similarities. The left panel shows an alignment of the GTPase 1 (G1) and the calcium-binding EF-hand 1 (EF1) domains, while the right panel displays an alignment of the EF-hand 2 (EF2) GTPase 2 (G2) domains.

**(B)** Schematic representation of the aligned G1-EF1 subdomain, indicating specific locations of zoomed-in insets shown in panels 1, 2, and 3 of Fig. S11C below.

**(C)** Schematic representation of MIRO1 post-translational modifications identified in publicly available datasets, illustrating acetylation (Ac) and phosphorylation (P) sites (panel 1). Panel 2 highlights functional mutations in human MIRO1 (blue labels), including a constitutively active mutation (P14V) and a dominant-negative mutation (T19N) in the G1 GTPase domain. Panel 3 depicts a mutation in the EF1 domain that disrupts calcium binding. Putative corresponding mutations in Arabidopsis MIRO1 are labeled in orange.

**(D)** Results of the structural alignment in (A) suggest a strong potential structural conservation of MIRO1 between humans and Arabidopsis despite moderate sequence conservation.

**
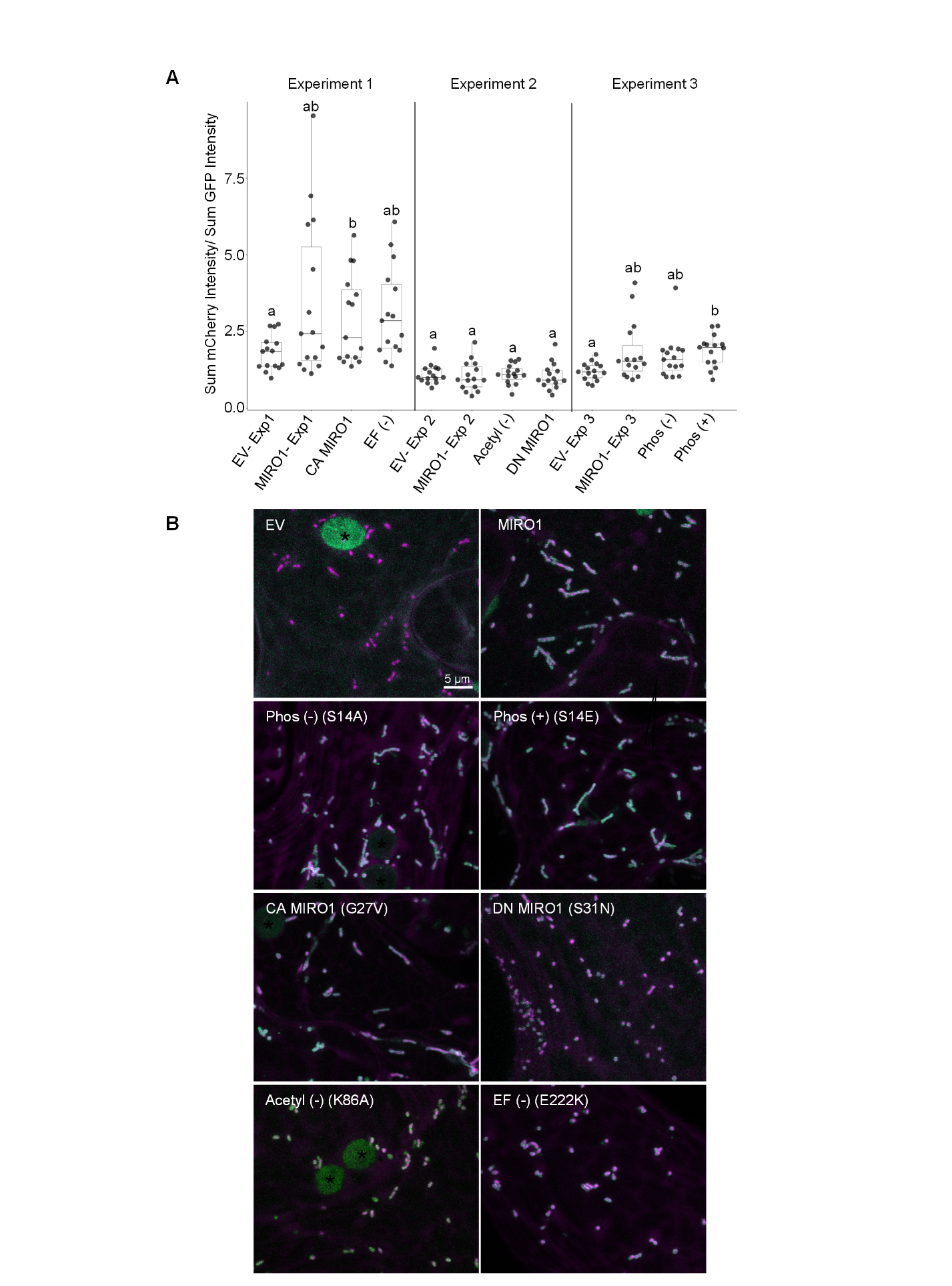
**

**Figure S12.** **Representative Images of Mitochondrial Lengths Quantified in Figure 9B**

**(A)** Ratio of mCherry to GFP intensity demonstrates comparable protein expression levels across MIRO1 mutant constructs in transient expression assays. Expression of mutant MIRO1 constructs was performed in 3 separate experiments each with an independent EV and wild type MIRO1 controls. In each experiment the mutant MIRO1 constructs show comparable protein expression to the relevant wild type MIRO1 controls. Letters denote statistically distinct groups: one-way wANOVA (EXP1) one-way ANOVAs (EXP2, EXP3); Tukey’s post-hoc test; n = 16 images; α = 0.05.

**(B)** Representative confocal images of mitochondrial morphology in transiently transformed tobacco leaves overexpressing an empty vector (EV, control) or N-terminally, GFP-tagged MIRO1 and GFP-tagged MIRO1 mutants, demonstrating mitochondrial outer membrane localization and mutation-dependent effects on mitochondria morphology. Chlorophyll autofluorescence indicated by *.

**Supplemental Files**

**Supplemental Movie 1. High light Treatment Reduces Mitochondria Movement, Related to Figure 2.**

Confocal microscopy was used to record 120 seconds of mitochondrial movement in epidermal cells exposed to standard light or high light for 1-, 6-, or 12-hours. Each frame (0.6 second frame time) was segmented, and movement of segmented mitochondria was tracked in ImageJ using TrackMate.

**Supplemental Movie 2. High light Treatment Increases Mitochondrial Fusion Related to Figure 2.**

Spinning disc confocal microscopy was used to observe instances of mitochondria fission and fusion in epidermal cells exposed to standard light or high light. Recordings 125 seconds in length with a frame time of 0.125 seconds per frame were recorded. Left recording is from standard light controls, middle and right recordings are from high light treated plants. Red arrows identify instances of mitochondrial fusion, blue arrow identifies instance of mitochondrial fission.

**Supplemental Movie 3. Tomogram of Mitochondria from Plants Treated with Standard Light, Related to Figure 3.**

Dual-axis electron tomography was used to visualize the ultrastructure of mitochondria from an epidermal cell exposed to standard light conditions. The tomogram reconstructed in ETOMO/IMOD corresponds to Figure 3 (A) and spans a volume of ~150 nm. Playback speed: 25 frames/sec.

**Supplemental Movie 4. 3D Reconstruction of Mitochondria from Plants Treated with Standard Light, Related to Figure 3.**

The tomogram from Supplemental Movie 3 was segmented using DeepMIB and rendered in 3D using Dragonfly 2. The reconstruction shows the ultrastructure of two mitochondria. Magenta indicates the outer envelope membrane; yellow is the inner membrane and cristae. The arrows at the corners show the rotation of the *x* (blue), *y* (red), and *z* (green) axes. Corresponds to Figure 3 B. Playback speed: 30 frames/sec.

**Supplemental Movie 5. Tomogram of Mitochondria from Plants Treated with High Light, Related to Figure 3.**

Dual-axis electron tomography was used to visualize the ultrastructure of mitochondria from an epidermal cell. The tomogram reconstructed in ETOMO/IMOD corresponds to Figure 3 (C, F, H) and spans a volume of ~150 nm. Playback speed: 25 frames/sec.

**Supplemental Movie 6. 3D Reconstruction of Mitochondria from Plants Treated with High Light, Related to Figure 3.**

The tomogram from Supplemental Movie 5 was segmented using DeepMIB and rendered in 3D using Dragonfly 2. The reconstruction shows the ultrastructure of three mitochondria. Magenta indicates the outer envelope membrane; yellow is the inner membrane and cristae. The arrows at the corners show the rotation of the *x* (blue), *y* (red), and *z* (green) axes. Corresponds to Figure 3 D, E, G, I. Playback speed: 30 frames/sec.

**Supplemental Table 1. Expanded Statistical Information, Related to all Figures and Supplemental Figures.**

**Supplemental Table 2. High Light and Fumarate Triggered Alterations in Epidermal Mitochondria Protein Abundances, related to Figure 6.**

**Supplemental Table 3. Primers used in this study.**

**Supplemental Table 4. Analyzed CCEM metabolites & LC-MS-MS parameters**
